# Recent Advances on Gallium-Modified ZSM-5 for Conversion of Light Hydrocarbons

**DOI:** 10.3390/molecules26082234

**Published:** 2021-04-13

**Authors:** Zhe Feng, Xin Liu, Yu Wang, Changgong Meng

**Affiliations:** School of Chemical Engineering, Dalian University of Technology, Dalian 116024, China; zfeng@mail.dlut.edu.cn (Z.F.); wangyu_200_2@dlut.edu.cn (Y.W.)

**Keywords:** light alkanes, ZSM-5, dehydrogenation, dehydroaromatization, olefins, Gallium

## Abstract

Light olefins are key components of modern chemical industry and are feedstocks for the production of many commodity chemicals widely used in our daily life. It would be of great economic significance to convert light alkanes, produced during the refining of crude oil or extracted during the processing of natural gas selectively to value-added products, such as light alkenes, aromatic hydrocarbons, etc., through catalytic dehydrogenation. Among various catalysts developed, Ga-modified ZSM-5-based catalysts exhibit superior catalytic performance and stability in dehydrogenation of light alkanes. In this mini review, we summarize the progress on synthesis and application of Ga-modified ZSM-5 as catalysts in dehydrogenation of light alkanes to olefins, and the dehydroaromatization to aromatics in the past two decades, as well as the discussions on in-situ formation and evolution of reactive Ga species as catalytic centers and the reaction mechanisms.

## 1. Introduction

Zeolites are a kind of aluminosilicate crystals with well-defined microporous systems, and are widely applied in catalysis, selective adsorption, ion exchange, etc. The basic structural unit in zeolite is TO_4_ tetrahedron (T = Si/Al), and these tetrahedrons are corner-sharing and interconnected by T-O-T bonds constituting cages and tetrahedrons [1]. In general, pure silica zeolites are comprised of SiO_4_ tetrahedrons where Si is +4 charged, and the framework is charge neutral. As Al (Ga) is +3 charged, the incorporation of AlO_4_ (GaO_4_) tetrahedrons into zeolite framework breaks the charge balance and extra-framework cations are necessary to neutralize the framework charges. A Brønsted acid site (BAS) is formed when a proton is used to balance the framework charge, while a Lewis acid site (LAS) is formed when an extra-framework metal cation is used. In principle, acidity of zeolite can be regulated by adjusting Si/Al ratio of the framework.

ZSM-5 is a kind of zeolites of MFI type framework and was first reported by Mobil Inc. The MFI framework is built with intersected straight channels of 5.3 Å × 5.6 Å and sinusoidal channels of 5.1 Å × 5.6 Å, both of which are 10-membered ring channels [2]. ZSM-5 is widely used as catalyst in chemical and petrochemical industry, for its intrinsic acidity, high surface area, superior porosity and thermal stability, etc. Furthermore, the intersected channel structure prevents the BAS from fast deactivation by coke deposition, enabling the excellent stability and catalytic selectivity [3].

In recent years, considerable research was applied for the development of novel and efficient catalysts and protocols for dehydrogenation and dehydroaromatization of light alkanes for the production of olefins and aromatics that are of higher economic value as feedstock to satisfy the growing need of chemical industry [4,5,6,7,8,9,10,11,12]. Catalytic dehydrogenation of alkanes is also proposed as the possible route for production of olefins with high selectivity, and thus, has the superiority over other technologies such as thermal cracking and stream cracking [13]. Further to these, as thermocracking is preferred, unmodified ZSM-5 catalysts always suffer from limited selectivity and high contents of undesired C_1_ and C_2_ in the product. As the same time, the strong acidity of ZSM-5 zeolites promotes coke deposition leading to deactivation of catalysts [14,15].

A variety of catalysts, such as Pt [16,17], CrO_x_ [11,18], MoC_x_O_x_ [19,20,21], amorphous carbon and carbon nanotubes [22,23,24], Ga oxide [25,26], etc., were found effective for non-oxidative dehydrogenation of light hydrocarbons. In Pt based bi-functional catalysts, Pt is the reaction species for dehydrogenation, while the acid sites of support account for isomerization and cyclization reactions. However, Pt is also capable of catalyzing scission of C-C bonds, leading to limited product selectivity to olefins [16,17,27]. Furthermore, these catalysts also suffer from carbon deposition and sintering of metal species in reaction conditions [28]. CrO_x_ was proposed effective for dehydrogenation in 1930s and is used in industry for butane dehydrogenation. Both carbon deposition in reaction conditions and diffusion of Cr species into the bulk support are the major sources of deactivation of CrO_x_ based catalysts [29]. The high cost of Pt and environmental issues associated with Cr have prompted the development of new catalysts. Mo-based catalysts are also used for converting of hydrocarbons. In Mo-based catalysts, the active Mo species are finely dispersed after calcination in form of MoO_x_ or MoO_x_C_y_, and the activity is also closely related to the type of support (such as Al_2_O_3,_ SiC, etc.) and preparation procedures. Fast deactivation, due to carbon deposition, is the major problem with Mo based catalysts [19,30]. When using ZSM-5 as support, the inherent acidity and channel structure of ZSM-5 were proposed to facilitate the dehydroaromatization [31,32].

Although Ga oxide-based catalysts appeared later, they have attracted much attention in recent years. Ga oxide-based catalysts still suffer from poor durability in reaction conditions [33]. Further investigations also showed that extra-framework metal species, such as Zn, Ga, etc. in zeolites, may have significant impact to the conversion of light alkanes. The catalytic performance of Zn and Ga modified ZSM-5 are found superior over others in light hydrocarbon dehyrdroaromatization [34,35,36,37]. However, the Zn species in modified ZSM-5 are volatile at high temperatures and in existence of hydrogen [38], and would get deactivated quickly [39,40]. Moreover, the reported yields of aromatics of Zn-modified ZSM-5 are much lower than those of Ga-modified ZSM-5 [41,42,43].

The synthesis and application of Ga-modified ZSM-5 can be traced back to the 1980s [33], and has been widely investigated for its efficiency in the conversion of light alkanes into aromatic chemicals (Cyclar process) [44]. Due to a lack of in-situ and operando experimental characterizations and theoretical techniques, the formation and evolution of the Ga species in reaction conditions and their contribution to the conversion of light alkanes, as well as carbon deposition, are not well-established. In this work, we focused on the recent progress on the synthesis and application of Ga-modified ZSM-5 as catalysts for dehydrogenation and dehydroaromatization of alkanes, as well as the discussions on in-situ formation and evolution of reactive Ga species as catalytic centers and the reaction mechanisms.

## 2. Preparation of Ga-Modified ZSM-5

Metal precursors and preparation procedures are vital for the successful fabrication of metal modified zeolites. Conventionally, Ga-containing salts [45,46,47,48,49,50] (gallium nitrate, etc.), trimethyl gallium [51], gallium chloride [52], gallium oxide [53,54], etc. can be used as precursors for fabrication of Ga-modified zeolites. The preparation method depends strongly on the chemical and physical properties of the selected metal precursor and the desired form of existence of Ga species in zeolite.

According to the Ga-species and the interaction with ZSM-5 framework, Ga-modified ZSM-5 for catalytic applications can be classified into 2 kinds, namely Ga isomorphous substituted ZSM-5 and ZSM-5 with extra-framework Ga species. In Ga isomorphous substituted ZSM-5, Ga take the framework position of Si or Al atoms in ZSM-5, forming BAS in the framework and these Ga species are framework Ga species. As for ZSM-5 with extra-framework Ga species, Ga species are formed from conversion of aggregated GaO_x_ particles from precursors on the external surface of the zeolite crystallites into Ga species capable to migrate into micropores of zeolite. Ga-modified ZSM-5 can be synthesized by chemical vapor deposition (CVD), incipient wetness impregnation, ion exchange and physical mixture with subsequent pre-treatment.

### 2.1. Isomorphous Substitution

Isomorphous substitution refers to the incorporation of trivalent Ga into the zeolite framework without changing the framework structure [55]. Generally, the incorporation of Ga into zeolite materials can be achieved during the crystallization process, normally in the presence of a suitable template, such as TPAOH [46,55,56], TBAOH [57], TBPOH [58], etc. In the product, Ga cations take the place of Si or Al in the framework of zeolite, which are highly dispersed and stable. It should be noted that, in Ga-substituted zeolites, Ga species only contribute to formation of BAS. Degalliation treatment, such as calcination, etc. would be necessary to convert these framework Ga species into extra-framework Ga species for catalytic applications, such as dehydrogenation and aromatization, etc. [41].

#### 2.1.1. Hydrothermal Crystallization

**Figure 1:**
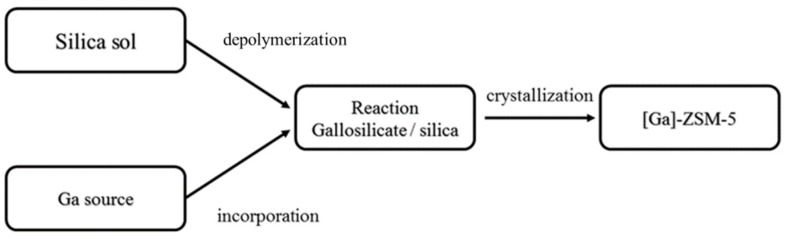
Hydrothermal synthesis process. Reprinted with permission from ref [59]. Copyright 1991 WILEY-VCH Verlag GmbH & Co. KGaA.

Hydrothermal crystallization is also the approach used to synthesis zeolites, including ZSM-5, etc. In this approach, the precursor of Ga, such as Ga_2_O_3_, Ga(NO_3_)_3_, etc. are added into synthesis gel for ZSM-5 crystallization, where Ga precursors are converted into [Ga(OH)_4_]^−^ ions. In the hydrothermal process, the [Ga(OH)_4_]^−^ ions in the gel are incorporated into the framework during the crystal growth in form of BASs. The hydrothermal crystallization process is shown in Figure 1.

The synthesis of Ga-modified ZSM-5 with hydrothermal crystallization has been reported [46,54,60,61,62,63,64,65,66,67,68,69]. Awate et al. reported synthesis of Ga-modified ZSM-5 from sodium gallosilicate gel using triethyl-n-butylammonium bromide as the structure-directing agents (SDA) [65]. Montes et al. reported the synthesis of TPA-MeNH_3_-[Ga_0.99_, Al_1.99_]-ZSM-5 in alkali-free gel, and investigated the activity and selectivity in dehydroaromatization of the samples. They showed that the activity and selectivity of the samples in dehydroaromatization can be adjusted by varying the reaction temperature, duration of calcination and steam pressure. The acidity and dehydrogenation activity of Ga-modified ZSM-5 can be adjusted by balancing the extra-framework Ga species and the number of BASs [66]. Raad prepared gallosilicates ZSM-5 zeolite using TPABr as SDA [70]. Nishi and Choudhary reported that Ga-modified ZSM-5, prepared using the hydrothermal method, was highly active in alkane dehydroaromatization but would be deactivated quickly [60,61,67]. Seed-induced hydrothermal crystallization of nanosized Ga-containing ZSM-5 was also reported [54,55].

#### 2.1.2. Recrystallization

Recrystallization is another approach for incorporation of Ga into zeolite framework. As zeolites can be considered products from the sequential condensation of silicic acids, hydrolysis is thus the reverse process and is in equilibrium with zeolite crystallization, recrystallization and growth. It should be noted that only a small portion of zeolite crystallite may be dissolved when it is in contact with a large amount of alkaline solution:

[SiO_2_]_n_(s) ↔ Si(OH)_4_ (1)

Shifting of this equilibrium during crystallization makes it possible to incorporation of the hydrated Ga species into the zeolite framework [71]. Due to the differences in stability and structure of GaO_4_ tetrahedron, with respect to those of SiO_4_ and AlO_4_ tetrahedrons, the incorporation of Ga into zeolite framework may induce framework disorder. Koslick reported the preparation of Ga ZSM-5 by recrystallization with prolonged crystallization time and observed migration of Ga species into framework positions [72].

### 2.2. Ga-Modified Zeolites

**Figure 2:**
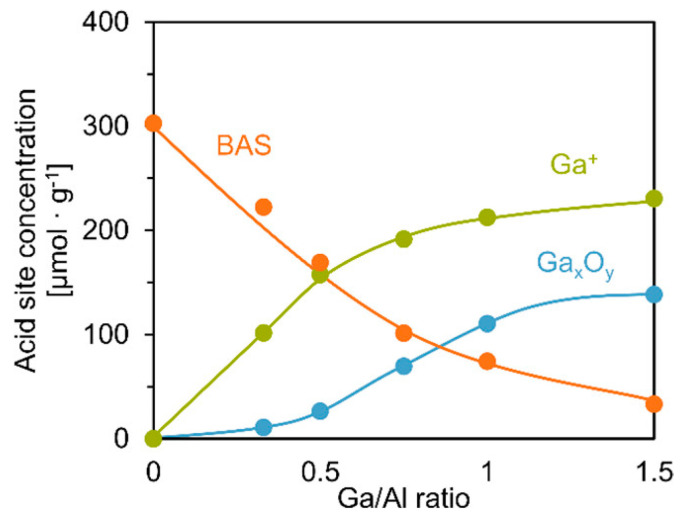
Concentrations of Lewis and Brønsted acid sites of catalysts as a function of the Ga/Al ratio. Reprinted with permission from ref [73]. Copyright 2018 American Chemical Society.

Different from isomorphous substituted zeolites with Ga in the zeolite framework, it is generally accepted that Ga precursors are converted into highly dispersed and positively charged species first. They may diffuse into the zeolite channels and take the place of protons, converting BAS to LAS in Ga-Modified Zeolites. Moritz reported that the amount of BAS in zeolite and GaO_x_ clusters on external surfaces decrease with the increase in LAS during the pretreatment (Figure 2). They also proposed that there was a synergy between BAS and LAS forming bi-functional reaction sites [73]. Conventionally, Ga-modified ZSM-5 can be synthesized through incipient wetness impregnation, ion exchange, chemical vapor deposition, etc.

#### 2.2.1. Incipient Wetness Impregnation

Incipient wetness impregnation is widely used for preparation of heterogeneous catalysts. For synthesis of Ga-modified ZSM-5, NH_4_-ZSM-5 or H-ZSM-5 samples are mixed with concentrated solution of precursors of Ga species, such as Ga(NO_3_)_3_, etc., until the solution is drawn into microchannels of zeolite by capillary action. Considering the larger volume compared to the pore size of ZSM-5, dispersion of hydrated Ga species into microchannels is still the subject of investigation. Samples of Ga-modified ZSM-5 fabricated in this way require further pretreatment to insure the dispersion of Ga species into the microchannels. Choudhary and Kazansky reported fabrication of Ga-modified ZSM-5 with Ga loading of 1.0, 3.0 and 5.0 wt% with incipient wetness impregnation [67,74,75].

#### 2.2.2. Ion Exchange

Ion exchange is another approach that is widely used for deposition of metal species onto support materials. The pH of the reaction solution and reaction temperature are two control factors that determine the success of the deposition. Ion exchanged can also be carried out in completely anhydrous condition. Due to the large volume, it is hard for hydrated Ga cations to reach the BAS in ZSM-5 channels. In this sense, most Ga precursors will be converted into microcrystals of GaO_x_ on external surface of zeolite during the subsequent calcination. This was supported by the reported scanning electron microscope (SEM) images of fresh samples. Therefore, it is also necessary to reduce GaO_x_ microcrystals into positively charged Ga species, dispersible into the zeolite channels, and enable them to take the place the proton and convert BAS into LAS. Karge provided a detailed description on the ion exchange for fabrication of Ga-modified zeolites [76]. Nowak et al. obtained Ga-modified ZSM-5 of different Ga loading by immersing NH_4_-ZSM-5 into 0.05 M aqueous solution of Ga(NO_3_)_3_ (pH = 3), with refluxing and stirring it at 373 K [77]. It should be noted that the Ga species can also be incorporated into the zeolite framework during the in-situ hydrothermal crystallization and liquid phase ion exchange [46].

Ga-modified ZSM-5 prepared by physical mixing of Ga_2_O_3_ and with HZSM-5 powder was also used in light hydrocarbon conversion reactions. The as-synthesized Ga-modified ZSM-5 required a very long induction time in the catalytic reactions, and pre-treatment may help to activate the sample [78]. Freeman et al. reported fabrication of Ga-modified ZSM-5 of 0.6~1.0 mm size by ball-milling the mixture of Ga_2_O_3_ and ZSM-5 powder, followed by pre-treatment [79,80].

#### 2.2.3. Chemical Vapor Deposition (CVD)

Contrary to Ga-modified ZSM-5, synthesized by ion exchange and incipient wetness impregnation, which require pre-treatment to achieve a reasonable dispersion of Ga species, extra-framework Ga species are well-dispersed in the samples synthesized by chemical vapor deposition (CVD) [75,81]. The volatile precursor like Ga(CH_3_)_3_ [39,82] and GaCl_3_ [52,83,84] are widely used as Ga precursor to obtain Ga-modified zeolites with CVD. Dried and calcined zeolite was mixed with Ga(CH_3_)_3_ or GaCl_3_ in a glove-box heated to 773 K for 24 h for dispersion of the decomposition products of Ga precursors into the zeolite channels. Reduction is also necessary to insure the dispersion of Ga species. Garcia-Sanchez reported the CVD preparation of Ga-modified ZSM-5 and Ga-modified MOR using Ga(CH_3_)_3_ as Ga precursor [81]. Phadke also prepared Ga-modified ZSM-5 with varying Ga/Al ratios through the anhydrous exchange of dehydrated H-MFI using GaCl_3_ as precursor [52,83].

#### 2.2.4. Pre-Treatment

**Figure 3:**
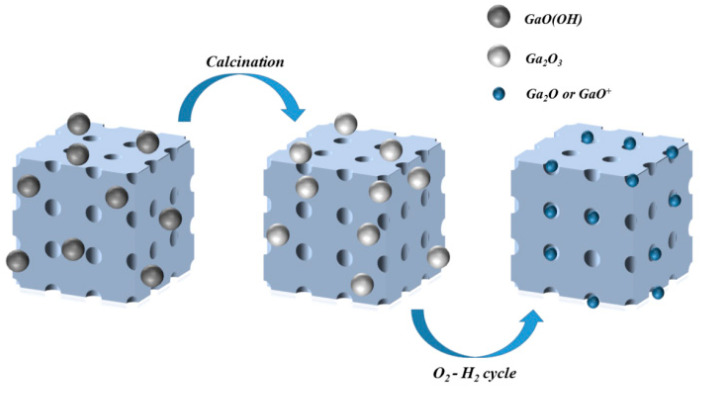
Changes in Ga species during pre-treatment.

The dispersion of Ga species in Ga-modified ZSM-5 samples synthesized by incipient wetness impregnation and ion exchange would be low before pre-treatment. A large portion of the Ga species are deposited on the external surface of the ZSM-5 as crystalline Ga_2_O_3_ after drying and calcination [85]. The reduction-oxidation cycles at temperatures of 637–823 K were found effective to promote redispersion and migration of Ga species in zeolite channels, where the highly-dispersed cationic Ga species are converted into reaction sites with reasonable stability [77,86]. With subsequent reduction-oxidation cycles, catalytic activity and dispersion of Ga species in Ga-modified ZSM-5 is significantly enhanced and the oxidation state of Ga species is reduced from +3 to +1. These low-valent Ga species (likely as volatile Ga_2_O) migrate into the zeolite channels, turning BAS into LAS [42,87,88]. Ausavasukhi proposed that water can react with the bulk Ga_2_O_3_ forming relatively well-dispersed GaO(OH) species that can further react with BASs to form [GaO]^+^ species (Figure 3) [89]. The variation of steam treatment and pH of the impregnation solution may help to enhance the dispersion of Ga species in zeolite channels. The pre-treatment of the freshly prepared catalyst, with hydrogen at elevated temperatures, was found to shorten the induction period and to increase activity [78]. Further investigations would be necessary to highlight the formation and evolution of Ga species in zeolite channels during the pre-treatment [90].

Ga_2_O_3_ + 2H_2_ → Ga_2_O + 2H_2_O (2)

In summary, Ga can be incorporated in ZSM-5 by different preparation methods, including hydrothermal synthesis, recrystallization, chemical vapor deposition, incipient wetness impregnation, ion exchange, etc. to form framework or extra-framework Ga species. The synthesis condition dependent performance of Ga-modified ZSM-5 suggests that the dispersion, formation and evolution of reactive Ga species would be highly reaction condition dependent. This calls for further investigations, either experimental or theoretical, to rational the knowledge on the synthesis approaches and reaction conditions with the performance of Ga-modified ZSM-5.

## 3. Reactive Species in Ga-Modified ZSM-5

### 3.1. Oxidation State of Ga Species in Ga-Modified ZSM-5

The structure and properties of extra-framework Ga species are more complicated, which are closely related to the preparation, choice of Ga precursor, pre-treatment and reaction conditions. Different precursors will lead to the deposition of Ga-species in different forms, framework or extra-framework, on the external surface or inside channels, and of different oxidation states (0, +1, +3). The oxidation state of framework Ga-species is +3. Extra-framework Ga species includes neutral bulk oxides, small particles, Ga-oxo clusters, Ga ions, etc. The oxidation state of Ga in neutral gallium oxide particles is +3, while the structure and oxidation state of Ga ions investigated are [Ga]^+^ (+1), [GaH_2_]^+^ (+3), [GaH]^2+^ (+3), [GaO]^+^ (+3), [Ga_2_O_2_]^2+^(+3), [Ga(OH)_2_]^+^ (+3), [GaHOH]^+^ (+3), etc. [46,50,52,91,92,93,94,95]. The redispersion of Ga species occurs during catalyst pre-treatment and even under reaction conditions. The pre-treatment of Ga-modified ZSM-5 with H_2_ at high temperatures leads to improved catalytic performance [35,96]. It is still debatable whether the H_2_ treatment produces only low-valent Ga centers (such as Ga^+^) or produces new active sites at the same time. Due to the existence of various Ga species and their interconversion in reaction conditions, there is still limited evidence to assign the enhanced catalytic performance to a certain type of Ga species of specific oxidation state.

### 3.2. Structure of the Active Site

Considerable research experimental and theoretical efforts have been devoted to unveil the “mystery” of active Ga species. The studies showed that [Ga]^+^, [GaH_2_]^+^ (+3), [GaH]^2+^ (+3), [GaO]^+^ (+3), [Ga_2_O_2_]^2+^ (+3), [Ga(OH)_2_]^+^ (+3), [GaHOH]^+^ (+3), GaO_x_ clusters, etc. may exist or coexist in Ga-modified ZSM-5. X-ray photoelectron spectroscopy (XPS), H_2_ temperature-programmed reduction (H_2_-TPR), ^71^Ga magic-angle spinning (MAS) nuclear magnetic resonance (NMR) spectroscopy, X-ray absorption near-edge structure (XANES), DRIFTS, Fourier transform infrared (FTIR), etc. have been used to characterize Ga species.

#### 3.2.1. Framework Ga Species

In the Ga-modified ZSM-5 prepared by hydrothermal crystallization and recrystallization, most of the Ga species are tetra-coordinated framework Ga-species. ^71^Ga magic angle spinning (MAS) NMR has been employed to characterize the framework Ga^3+^ species and the corresponding signal was found in the range 150–160 ppm [66,97]. X-ray diffraction (XRD) and FTIR spectra validated the existence of framework Ga species [55]. Framework Ga species is hard to be reduced and cannot be characterized by H_2_-TPR in situ [98].

#### 3.2.2. Extra-Framework Ga Species

##### Gallium Oxide Particles

As neutral Ga oxide is active for dehydrogenation of alkanes, they were proposed to account for the enhanced dehydrogenation activity and aromatics selectivity of Ga-modified ZSM-5, as compared with H-ZSM-5 [25,97,99,100]. Michorczyk investigated the propane dehydrogenation catalyzed by Ga_2_O_3_ in CO_2_ atmosphere. They showed that Ga_2_O_3_ fabricated by CVD are highly active for dehydrogenation, and CO_2_ enhances the initial activity of Ga_2_O_3_. Catalytic performance and product selectivity of Ga_2_O_3_ can be optimized further by deposition onto zeolite and other supports [99,101]. Shao studied the performance of several Ga_2_O_3_ catalysts on different supports for propane dehydrogenation. They showed that Ga_2_O_3_/ZSM-5 (5% Ga_2_O_3_ loading) exhibits the highest catalytic activity, which can be attributed to the well-dispersion and high dehydrogenation efficiency of cationic Ga species (Ga^δ+^, δ < 2) [53]. Xu and Shen et al. showed that dealumination can promote the propane dehydrogenation performance of Ga_2_O_3_/HZSM-5 by reducing the intermediate acid sites on HZSM-5 and GaO_x_ [26,102]. Strong acid sites may improve the catalytic activity, but can also lead to quickly catalyst deactivation. In this regard, dealumination is resorted to control Si/Al ratio to reach a good balance between catalytic activity and durability of the catalyst [26,102].

Gallium oxide particles or clusters, located in the channels and/or external surface of the zeolite were usually produced by ion exchange or incipient wetness impregnation [50,103,104,105,106]. The potential existence of octahedral coordinated Ga^3+^ or highly dispersed Ga_2_O_3_ can be identified by the chemical shift in the range from −7 to 24 ppm on ^71^Ga MAS NMR, and the appearance of reductive peaks in H_2_-TPR from 670 to 1170 K [45,50,58,70,103,107,108].

**Figure 4:**
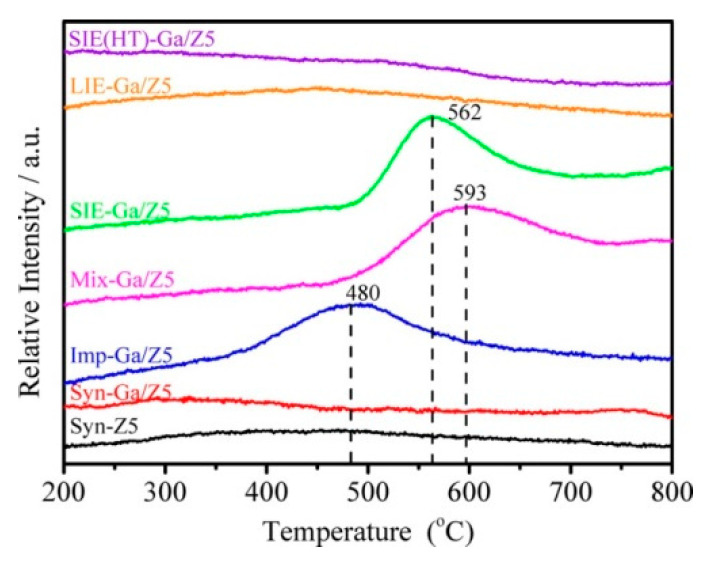
H_2_-TPR profiles of Ga/ZSM-5 prepared via different methods. Reprinted with permission from ref [46]. Copyright 2019 American Chemical Society.

However, the reaction kinetics and thermogravimetric (TG) analysis show that the catalytic performance of Ga-modified ZSM-5 is superior over Ga_2_O_3_ clusters [46,108]. The increase of Ga loading may lead to decrease of BAS and enhanced dehydrogenation activity of Ga-modified ZSM-5. In this sense, Ga species associated with BAS are also active for dehydrogenation [42]. XANES [82] and DRIFT [109] spectra also evidenced the presence of multiple Ga species, rather than Ga_2_O_3_ clusters after reduction in hydrogen (Figure 4).

##### Cationic Ga Species

Meitzner [96] and Hensen [82] found that the active Ga species may be in either trivalent oxidized or reduced hydride form to balance framework charge. These positively charged reduced Ga species can evolve into low-valent species through reductive elimination, or become oxidized through a reaction with oxygen-containing species, such as water and alcohol.

**Figure 5:**
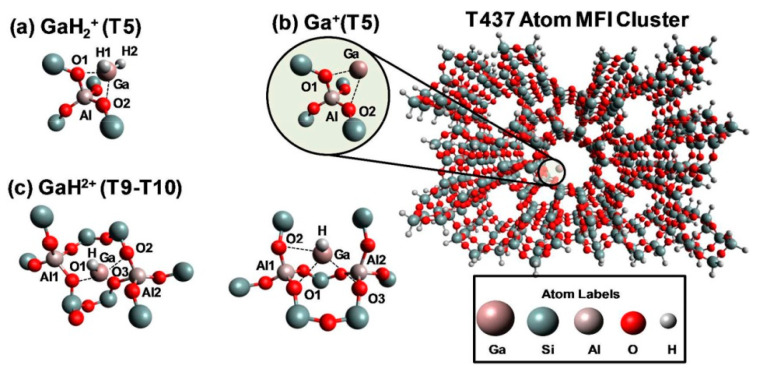
View along the [10] axis of T437 atom MFI structure used to model Ga-exchanged sites in Ga-modified ZSM-5. (**a**) [GaH_2_]^+^; (**b**) Ga^+^; (**c**) [GaH]^2+^. Reprinted with permission from ref [94]. Copyright 2018 American Chemical Society.

**Figure 6:**
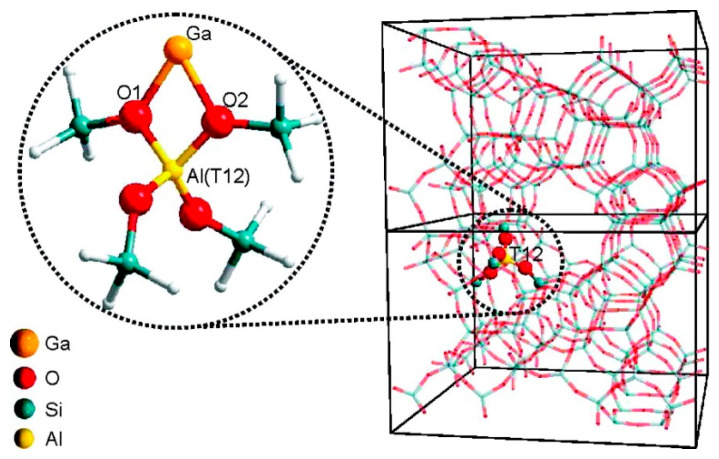
Structure of MFI zeolite and Ga^+^Z^−^ cluster model used in the DFT calculations. Reprinted with permission from ref [114]. Copyright 2008 American Chemical Society.

Ga^+^ was proposed as the reaction center for alkanes dehydrogenation in many reports [73,85,87,89,92,95,109,110,111,112]. Hensen [82] and Rane [112] et al. investigated the reduction of Ga species after CVD with FTIR and XANES. They showed that the [Ga(CH_3_)_2_]^+^ species reacts with BAS forming Ga^+^ and [GaH_2_]^+^ as LAS. The reaction kinetics showed that a large amount of propylene is produced only on Ga^+^. The XANES data showed that the reduction of Ga-alkyl species starts at ~640 K and with formation of Ga^+^ species [82]. Early theoretical studies suggest that the Ga^+^ is thermodynamically favored under reaction conditions at 823 K when a T8 cluster model was used to mimic zeolite [113]. However, the recent investigations showed that the observed edge energy shift may be due to the change in coordination of Ga^3+^ under reaction conditions, rather than the reduction of Ga^3+^ to Ga^+^ [114]. Therefore, XANES cannot be used to distinguish Ga^+^ from [GaH_2_]^+^ [73]. Kazansky et al. revealed that Ga^+^ species are extremely unstable. They proposed that Ga^+^ species tend to react with trace amount of water or framework oxygen atoms to form [Ga(OH)_2_]^+^ or [GaO]^+^ or even Ga_2_O_3_ clusters even when cooled in hydrogen atmosphere [75]. Mansoor et al. found that the barrier for C-H activation is higher on Ga^+^ than those on [GaH_2_]^+^ or [GaH]^2+^(Figure 5) [94]. The theoretically proposed Ga^+^ structure is also shown in Figure 6 [114].

Cationic GaH_x_ was also identified experimentally and theoretically in conversion of light hydrocarbons [75,109,114,115,116,117,118]. The existence of tetrahedral Ga^3+^ of [Ga(OH)_2_]^+^ or [GaH]^2+^ attached to BAS can be identified by ^71^Ga MAS NMR resonance signal at ~150 ppm [58]. Formation of cationic Ga^+^ and [GaH]^2+^ species from reduction of the neutral gallium oxide particles can be evidenced by the broad reductive peak around 700 K in H_2_-TPR of Ga modified ZSM-5 [51]. Arnaldo et al. investigated the dehydrogenation of propane over Ga-modified ZSM-5, and proposed according to the simultaneously increased propane conversion, H_2_ production and H_2_/propane ratio that reaction site may form dynamically, when propane is in contact with the Ga species [88]. Krishnamurthy et al. proposed that [GaH]^2+^ as the dominant Ga species when the Ga/Al ratio is low, while [GaH_2_]^+^ becomes the active species when the Ga/Al ratio is higher. They also showed that the propane conversion and the aromatics selectivity is highest when Ga/Al = 0.5 [42]. Phadke et al. prepared Ga-modified ZSM-5 with Ga/Al = 0.1~0.7 with CVD. Combining experimental and theoretical efforts, they proposed that cationic [Ga(OH)_2_]^+^ and [Ga(OH)]^2+^ are the Ga species when the Ga/Al ratio is lower than 0.3 and they are reduced to [Ga(OH)H]^+^ after hydrogen treatment. The difference of species after reduction is closely related to the distance between framework BAS and partial pressure of hydrogen. [Ga(OH)H]^+^ may react with the proton of neighboring BAS forming [GaH]^2+^ [52,83]. Ausavasukhi et al. studied the ethane dehydrogenation reaction over various Ga species (including Ga_2_O_3_, [GaO]^+^, [GaH_2_]^+^, Ga^+^, etc.) on HZSM-5. They proposed that [GaH_2_]^+^ is highly active in the dehydrogenation of ethane, but this species will decompose in the absence of H_2_ [89].

**Figure 7:**
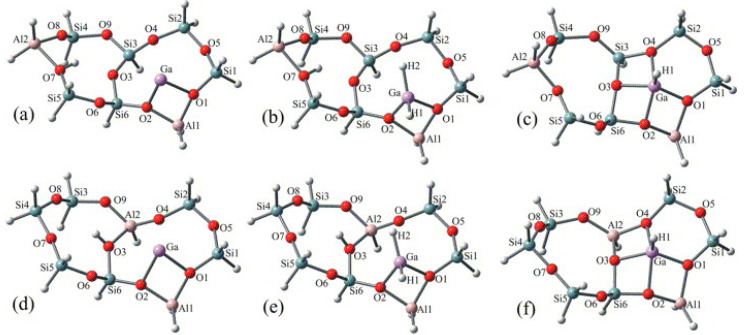
Optimized structures of potential Ga species in Ga-modified ZSM-5. (**a**) Ga^+^ Z_d_, (**b**) [GaH_2_]^+^ Z_d_, (**c**) [GaH]^2+^ Z_d_, (**d**) Ga^+^ Z_s_, (**e**) [GaH_2_]^+^ Z_s_, and (**f**) [GaH]^2+^ Z_s_ clusters. Reprinted with permission from ref [113]. Copyright 2006 Elsevier.

Joshi et al. showed that [GaH]^2+^ would be stable with LAS with two adjacent framework Al sites and would be active for ethane dehydrogenation. They also showed that there is a correlation between the dehydrogenation barriers with the distance between framework Al sites [119]. Pereira et al. investigated light alkane dehydrogenation over extra-framework Ga species and their proposed structures of these Ga species are in Figure 7 [120]. Frash et al. investigated the role [GaH_2_]^+^ and [GaO]^+^, and proposed that [GaH_2_]^+^ is a potential reaction site for dehydrogenation and [GaO]^+^ is hard to get regenerated in reduction conditions [117]. However, Rodrigues et al. proposed that [GaH_2_]^+^ is not the only active site of alkane dehydrogenation according to results from pyridine IR, in-situ DRIFTS and catalytic performance [87].

Kazansky et al. used H_2_ and N_2_O to oxidize Ga^+^ in ZSM-5 at different temperatures. They showed that good dispersion of [GaO]^+^ can be achieved. The proposed mechanism is as shown in Equations (3)–(5) [109]:

Ga_2_O_3_ + 2 H_2_ → Ga_2_O + 2 H_2_O (3)

Ga_2_O + 2 ZO^−^ ··· H^+^ → 2 ZO^−^ ··· Ga^+^ + H_2_O (4)

2 ZO^−^ ··· Ga^+^ + O_2_ → 2 ZO^−^ ··· [GaO]^+^ (5)

**Figure 8:**
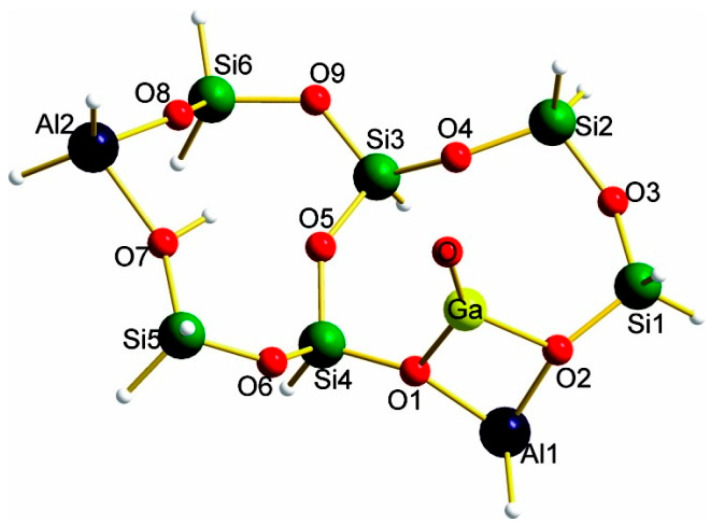
Optimized structure of the [GaO]^+^ ion stabilized in the cluster model representing ZSM-5 zeolite. Reprinted with permission from ref [116]. Copyright 2007 American Chemical Society. To this end, the existence and reactivity of various Ga species have been evidenced experimentally and theoretically. However, it is still difficult to rationalize the formation evolution of these Ga species, and their contribution to the conversion of light alkanes in dehydrogenation to olefins to the observed catalytic performance. It may call for further investigations with in-situ and operando techniques.

The existence of cationic [GaO]^+^ species in Ga-modified ZSM-5 can be identified by ^71^Ga MAS NMR resonance signal at ~55 ppm [104,121]. The characteristic peaks of the reduction of extra-framework [GaO]^+^ species in H_2_-TPR were observed in the range of 990 K to 1014 K [57,70,91,107]. The observed decrease of Ga coordination symmetry in Ga-modified ZSM-5 in ^71^Ga quadrupolar Carr-Purcell-Meiboom-Gill NMR (QCPMG) spectra can also be ascribed to formation Ga species in the form of [GaO]^+^ or hydrated cationic [GaO]^+^ [122,123]. Clustering of [GaO]^+^ to [Ga_2_O_2_]^2+^ was also proposed in Ga-modified ZSM-5 and may be more active than Ga^+^ in dehydrogenation of light alkanes [93,113,124]. Faro et al. investigated the correlation between the coordination number of the second Ga coordination sphere and the Ga^3+^ content of all partially reduced catalysts with in-situ XANES and EXAFS. They showed that GaO_x_ was converted into binuclear Ga species at the early stage of reduction. They also proposed that formation of these charged species can be explained with the acidity of ZSM-5. The strong LASs promote the formation of reduced Ga species [125]. Fang et al. showed that isomorphic substitution promoted dispersion of Ga precursors into zeolite channels forming extra-framework [GaO]^+^ species. They confirmed the existence of this [GaO]^+^ species with pyridine IR and catalytic test. [126] Lai and co-workers synthesized Ga-impregnation in mesoporous ZSM-5 to enhance migration of hydrated Ga ions to the zeolite cavity [GaO]^+^-BAS bi-functional sites [50]. Hensen [124] and Ausavasukhi [89] showed that the addition of water had a positive effect on the dehydrogenation of alkanes through the regeneration of [GaO]^+^ cations. Xiao et al. used formic acid in impregnation and in-situ treatment to promote the dispersion of Ga species and the formation of [GaO]^+^ species. These [GaO]^+^ would replace the protons of BAS, forming strong LAS. The synergy between LAS and BAS may account for the observed catalytic performance in dehydrogenation [103,106]. Uslamin et al. found that ZSM-5 with 4% Ga loading was stable after ten regeneration cycles at high temperature, and proposed that [GaO]^+^ species was highly stable in Ga/ZSM-5 [127]. Early theoretical studies also suggested that [GaO]^+^ stabilized at the ion exchange site of zeolite are highly reactive in initiating methane C-H bond cleavage at low activation energy [128]. Pidko et al. found that, while [GaO]^+^ is highly reactive, it is hard to get regenerated and cannot be considered as reactive species (Figure 8) [116].

## 4. Mechanisms of Alkanes Conversion over Ga-Modified ZSM-5

Light olefins are the raw materials for production of polymers and olefin derivatives [129]. Among various catalysts, Ga-modified ZSM-5 was found efficient and highly selective in the production of light olefins and aromatics from light alkanes through dehydrogenation. In reaction conditions, dehydrogenation of light alkanes may couple with thermocracking, oligomerization and cyclization, forming olefins and aromatics. [130] Several extra-framework Ga species, such as [Ga]^+^, [GaH_2_]^+^ (+3), [GaH]^2+^ (+3), [GaO]^+^ (+3), [Ga_2_O_2_]^2+^ (+3), [Ga(OH)_2_]^+^ (+3), [GaHOH]^+^ (+3), GaO_x_ clusters, etc., were proposed as the active sites in dehydrogenation with Ga-modified ZSM-5. Considerable research attention, both experimental and theoretical, has been devoted continuously to identifying these extra-framework Ga species and their roles in dehydrogenation of light alkanes to alkenes [111,114,115,118,122,129].

Both dehydrogenation and dehydrocyclization of light alkanes initiates with the activation and dehydrogenation of the C-H bonds of alkanes [131]. The dehydrogenation of alkanes starts with the “H” transfer, and depending on the charge on the formed hydrocarbon species, the proposed mechanism can be classified into “Carbenium activation” and “Alkyl activation”. The “Alkyl activation” pathway involves the formation of Ga-C bonds. The “Alkyl activation” pathway, also known as the “three-step mechanism”, includes (1) activation of C-H bond (R^δ−^-H^δ+^); (2) alkyl abstraction by Ga with the formation of alkyl-Ga species; (3) formation of alkenes from the alkyl group bound to Ga-species. A one-step “Concerted mechanism” was also proposed by Perrira, where the elimination of olefins and H_2_ formation take place simultaneously [132].

**Figure 9:**
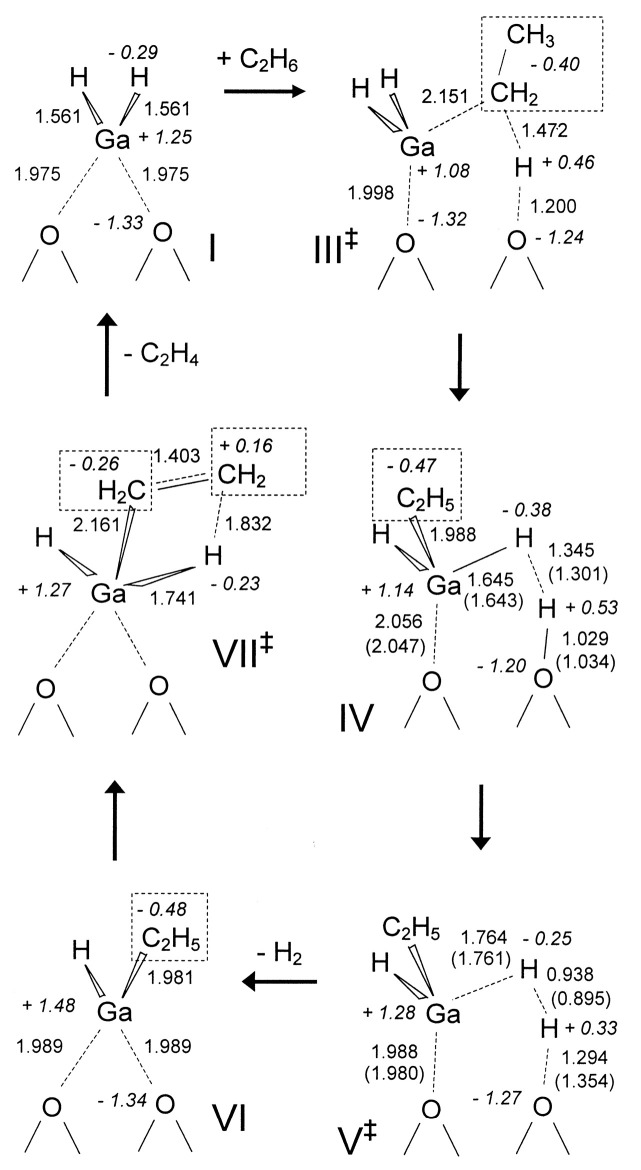
Reaction route for “alkyl” activation of ethane on [GaH_2_]^+^. Reprinted with permission from ref [117]. Copyright 2000 American Chemical Society.

**Figure 10:**
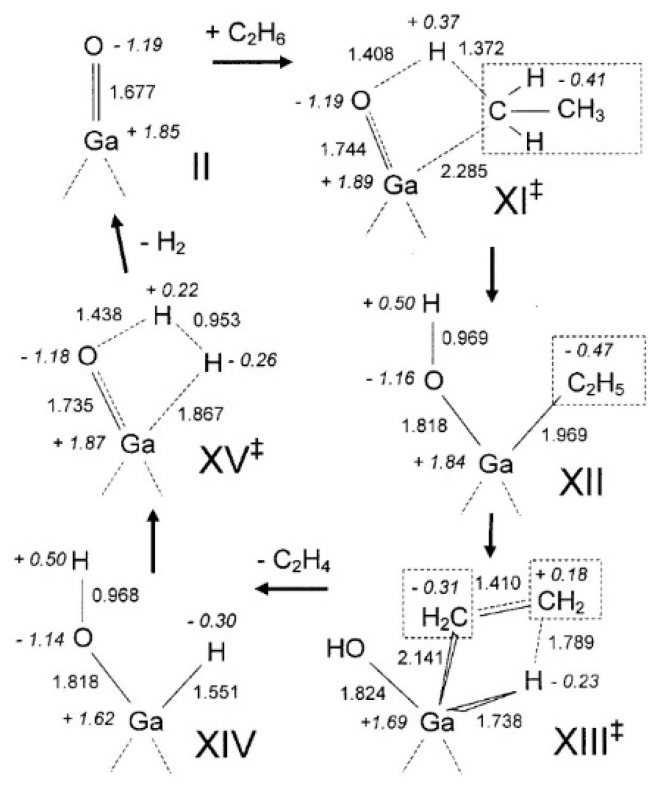
Reaction route for “alkyl” activation of ethane on the adsorbed gallyl ion. Reprinted with permission from ref [117]. Copyright 2000 American Chemical Society.

**Figure 11:**
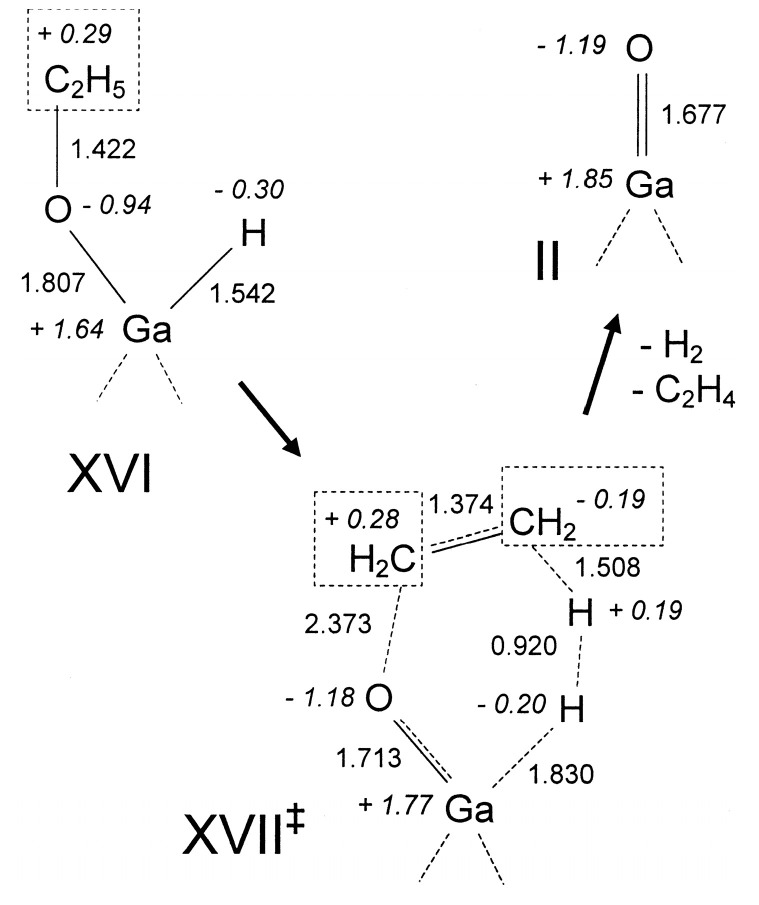
Regeneration of the adsorbed gallyl ion after “carbenium” activation of ethane. Reprinted with permission from ref [117]. Copyright 2000 American Chemical Society.

**Figure 12:**
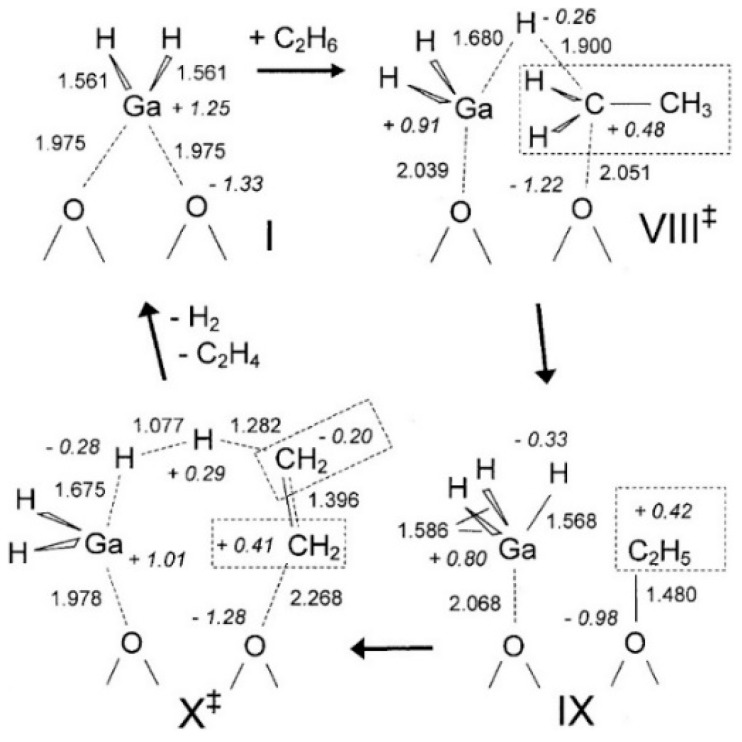
Reaction route for “carbenium” activation of ethane on [GaH_2_]^+^. Reprinted with permission from ref [117]. Copyright 2000 American Chemical Society.

Frash and van Santen investigated dehydrogenation of ethane in [GaH_2_]^+^ and [GaO]^+^ species in Ga-modified ZSM-5 through quantum chemical calculations. In their work, the calculated activation energy barriers over [GaH_2_]^+^ species along the “Alkyl activation” route were in good agreement with the experimental values (Figure 9). Due to the high barrier for regeneration, [GaO]^+^ species was not considered an active species (Figure 10 and Figure 11). Alkoxy-like intermediates were formed after C-H (R^δ+^-H^δ-^) activation along the “Carbenium activation” pathway. This pathway is more difficult because of the poor stability of the alkyl cation (Figure 12) [117].

**Figure 13:**
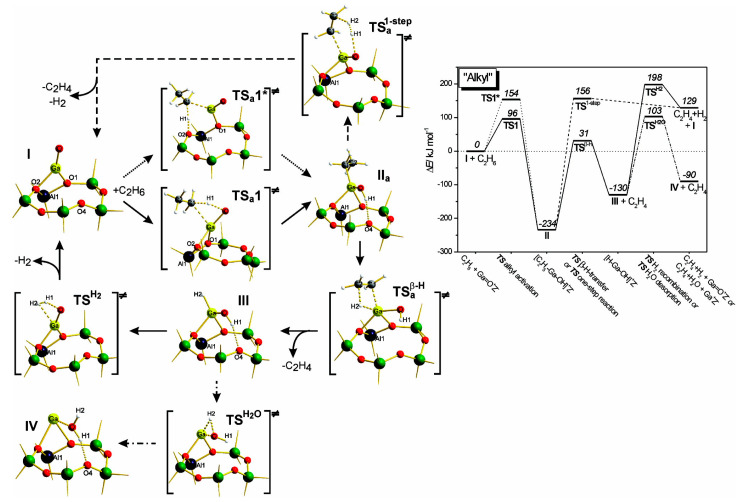
Reaction paths for the “alkyl activation” mechanism of ethane dehydrogenation over gallyl ion in ZSM-5 zeolite. Reprinted with permission from ref [116]. Copyright 2007 American Chemical Society.

Pidko studied ethane dehydrogenation over [GaO]^+^ using an 8T cluster with density functional theory calculations. After the heterogeneous dissociation of ethane on Ga species through “Alkyl activation” pathway, with a low activation energy barrier, [C_2_H_5_-Ga-OH]^+^ is formed as a very stable intermediate. Further reactions of ethane on Ga^+^ take places through the “Carbenium activation” mechanism forming [H-Ga-O-C_2_H_5_]^+^. They also showed that it is hard to regenerate the [GaO]^+^ according to the reaction thermodynamics and kinetics (Figure 13) [9].

**Figure 14:**
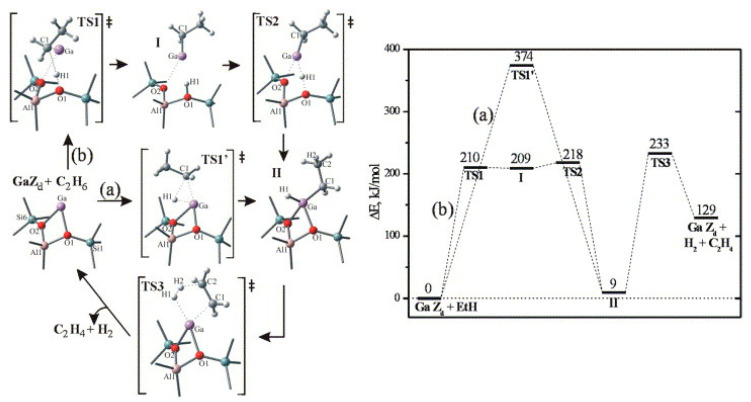
Homolytic (**a**) and heterolytic (**b**) “alkyl” pathways of ethane dehydrogenation over Ga^+^ Z_d_ site. Reprinted with permission from ref [113]. Copyright 2006 Elsevier.

Pidko compared the stability of Ga^+^, [GaH_2_]^+^ and [GaH]^2+^ species and the possible pathways of alkane dehydrogenation over these Ga species by theoretical calculations. They proposed that Ga^+^ is active species in the “Alkyl activation” mechanism with Ga as the acceptor of alkyl groups (Figure 14) [113].

**Figure 15:**
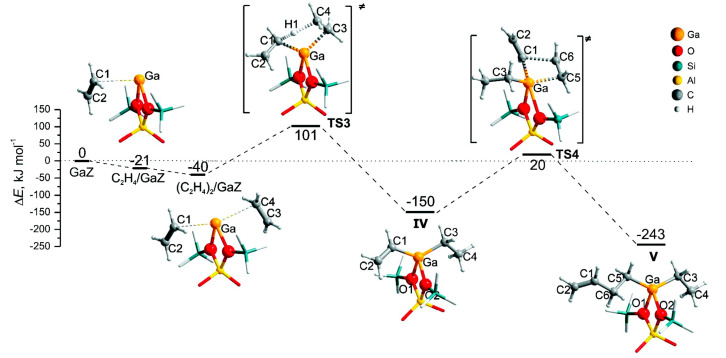
Ethylene dimerization over Ga^+^Z^−^. Reprinted with permission from ref [114]. Copyright 2008 American Chemical Society.

They also investigated ethane dehydrogenation and ethylene dimerization on extra-framework Ga^+^ with a 12T cluster model (Figure 15). They showed that catalytic dehydrogenation of ethane over extra-framework Ga^+^ in ZSM-5 may experience a reaction barrier of ~224 kJ/mol with formation of [Ga^3+^(H^−^)(C_2_H_5_^−^)]^+^ as the primary intermediate. The subsequent dimerization may proceed with formation of vinyl and ethyl groups, which are linked to cationic Ga species from two adsorbed C_2_H_4_. Further reaction between ethyl and vinyl groups leads to formation of butene. This mechanism is similar to classic C-C coupling catalyzed by BAS with carbenium as intermediate and transition metal catalyzed anionic olefin oligomerization [114].

**Figure 16:**
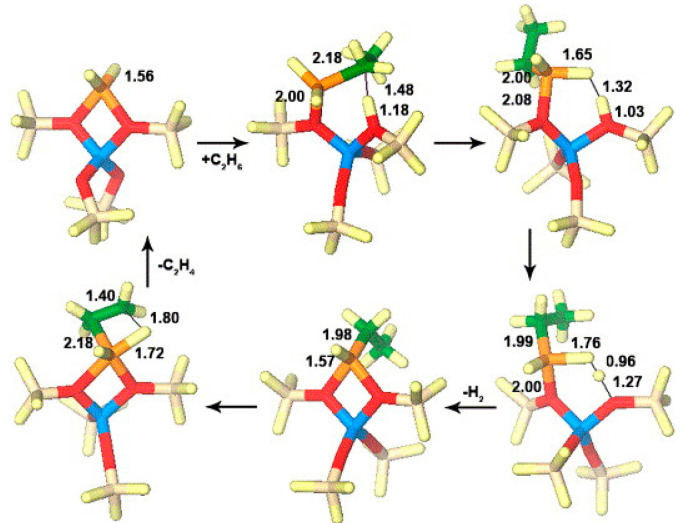
Ethane dehydrogenation reaction through the 3-step mechanism. Reprinted with permission from ref [132]. Copyright 2005 Elsevier.

**Figure 17:**
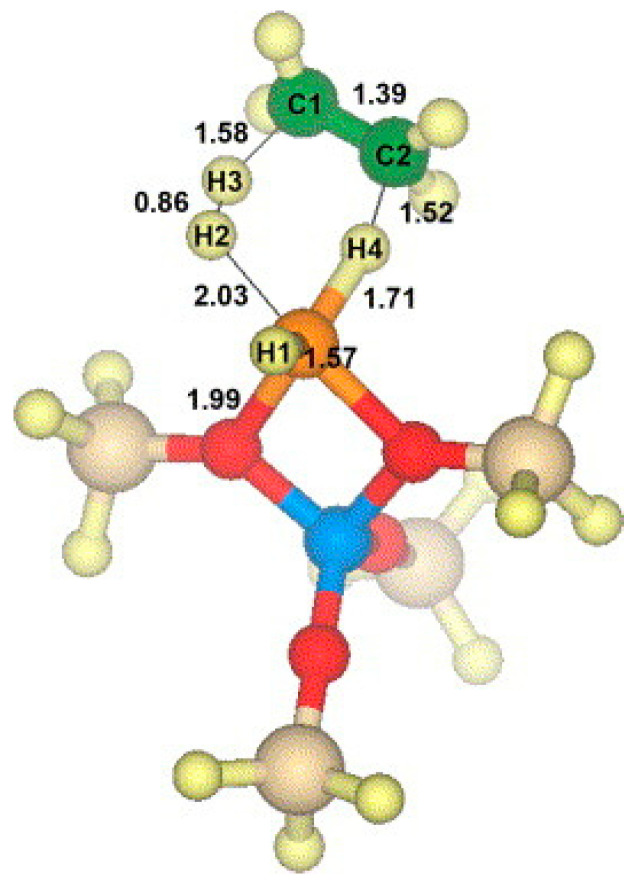
Transition state for the dehydrogenation reaction of ethane through the concerted mechanism. Reprinted with permission from ref [132]. Copyright 2005 Elsevier.

**Figure 18:**
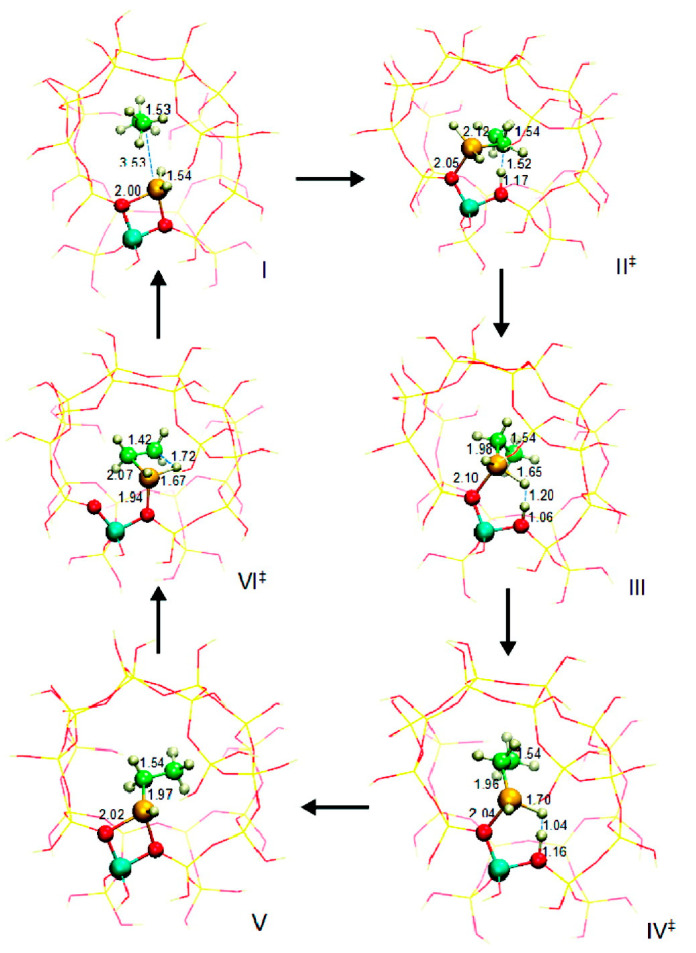
Dehydrogenation reaction of ethane through the three-step mechanism computed with GaH_2_ species and T22 cluster (B3LYP/6-31G**). Reprinted with permission from ref [133]. Copyright 2008 American Chemical Society.

**Figure 19:**
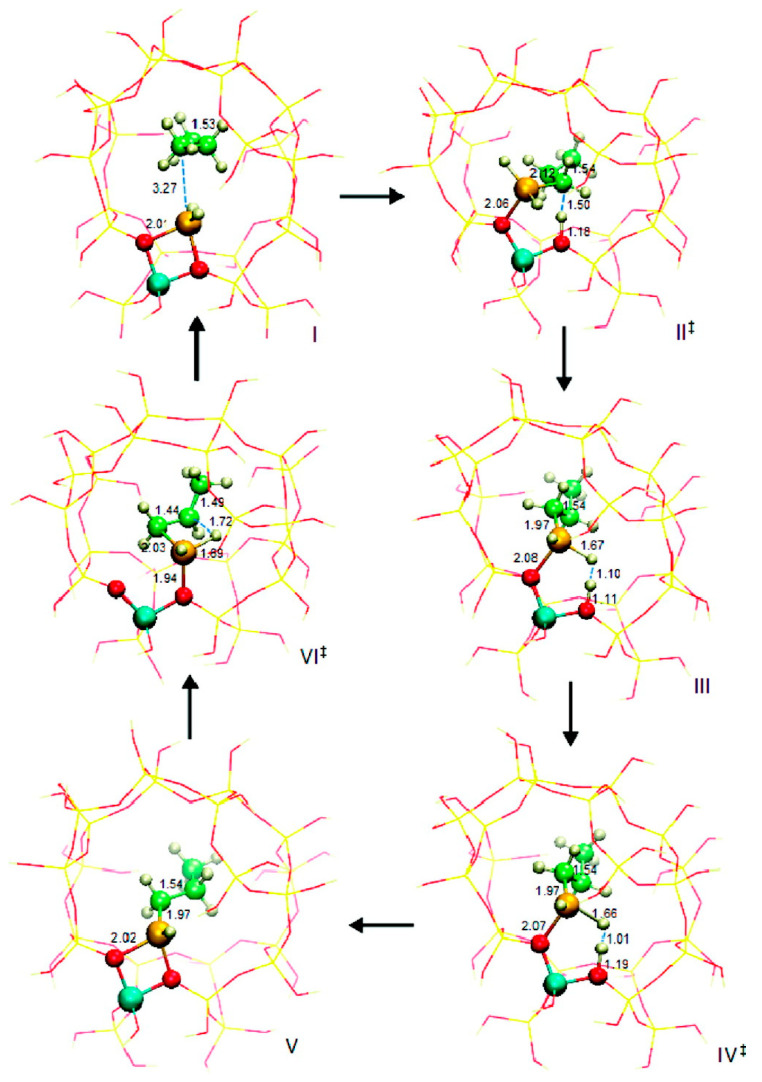
Dehydrogenation reaction of propane through the three-step mechanism (channel 1) computed with GaH_2_ species and T22 cluster (B3LYP/6-31G**). Reprinted with permission from ref [133]. Copyright 2006 American Chemical Society.

Perrira et al. investigated the mechanism for ethane dehydrogenation through “alkyl activation” and “concerted mechanism”, with density functional theory-based calculations and a 5T cluster model with [GaH_2_]^+^ ion as the active site (Figure 16 and Figure 17). They showed that “concerted mechanism” would be favored, though the barriers for reactions on “Alkyl activation” pathways are lower, considering the effects of the zeolite framework as a large support. As the “Alkyl activation” was very sensitive to the pore structure, the cluster model was further optimized and expanded to a 22T cluster (Figure 18 and Figure 19). They showed that ethane dehydrogenation is plausible through “Alkyl activation” mechanism over [GaH_2_]^+^ species. The “Alkyl activation” would compete with “concerted mechanism” for dehydrogenation of propane and n-butane, while “concerted mechanism” is favored for isobutane. These results suggested that a “concerted mechanism” would be the dominant mechanism for the substrates of the large volume [120,132,133].

**Figure 20:**
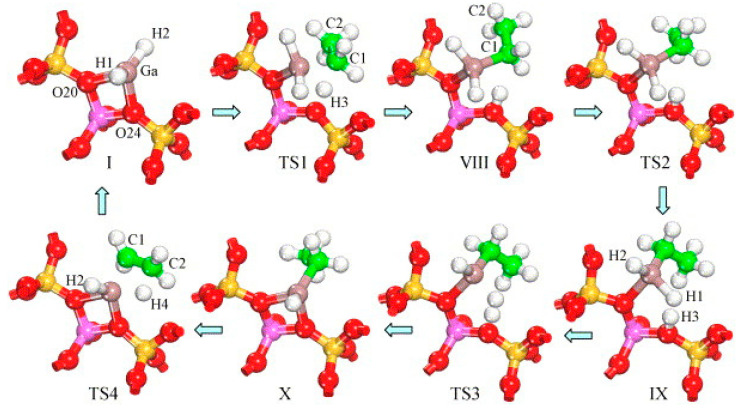
Molecular transformations during dehydrogenation of ethane on Z^−^[HGaH]^+^ site (terminal SiH_3_ are not shown for clarity). Reprinted with permission from ref [119]. Copyright 2005 Elsevier.

**Figure 21:**
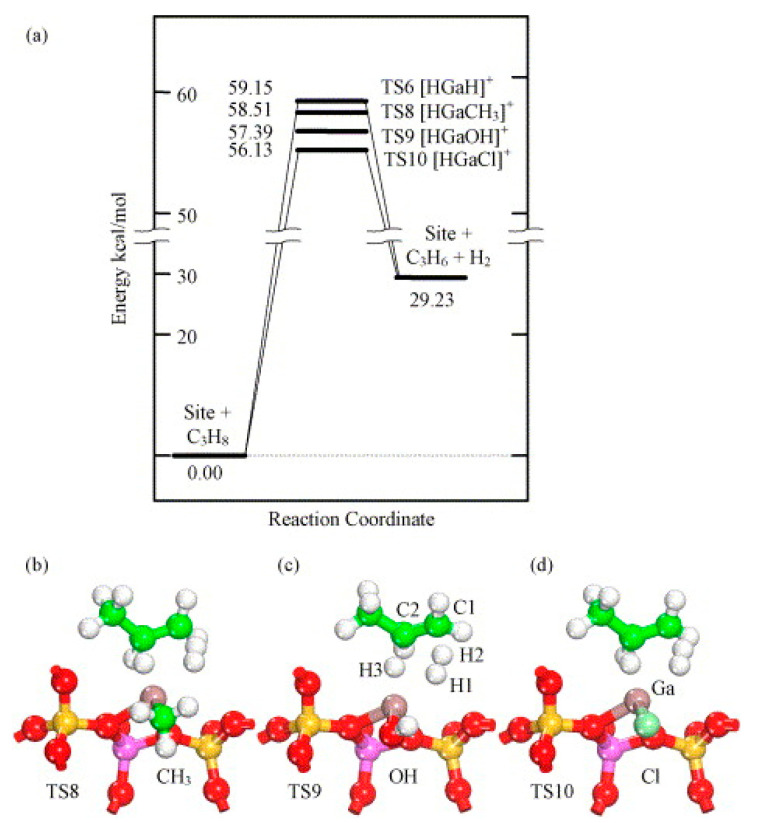
Direct (one-step) dehydrogenation of light alkanes on the Z^−^ [HGaH]^+^: (**a**) reaction path; transition state geometries for (**b**) X = CH3, (**c**) X = OH and (**d**) X = Cl. Reprinted with permission from ref [119]. Copyright 2005 Elsevier.

**Figure 22:**
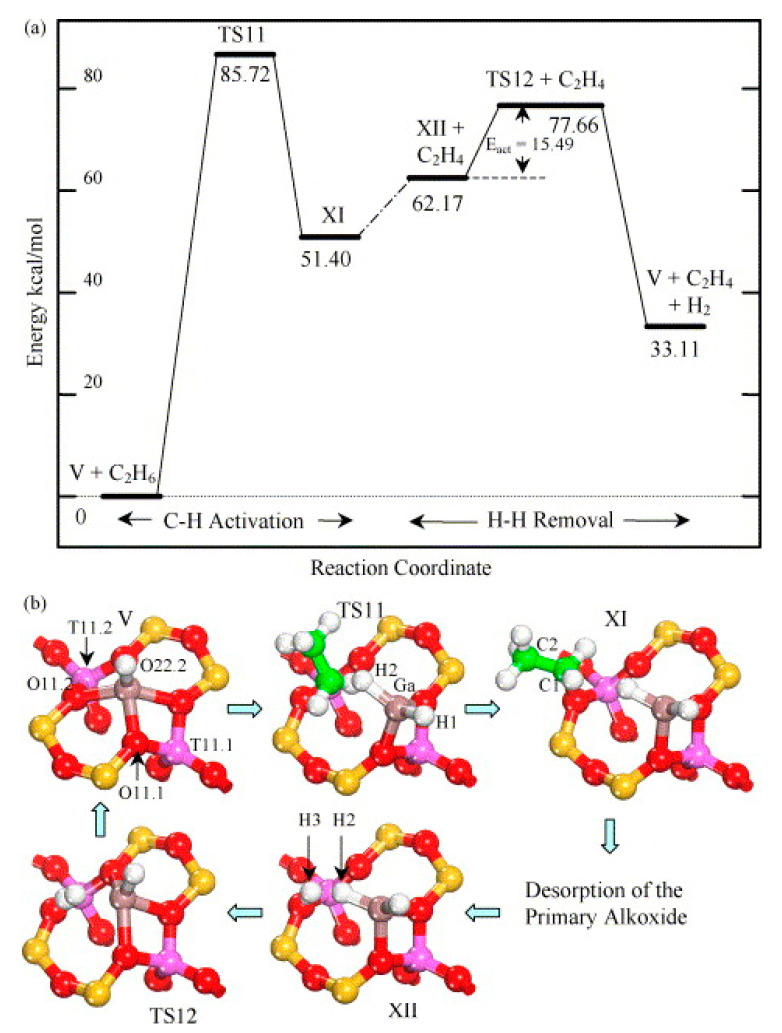
Carbenium activation path of ethane dehydrogenation on Z^2−^GaH^2+^ species(V) in six-membered ring: (**a**) reaction path consisting two-distinct steps and (**b**) geometry transformations during dehydrogenation path. Reprinted with permission from ref [119]. Copyright 2005 Elsevier.

Joshi et al. proposed that the reaction site in Ga-modified ZSM-5 was composed of Ga species and the adjacent BAS, and light alkane dehydrogenation may take place through one-step dehydrogenation mechanism (Figure 20, Figure 21 and Figure 22) [119]. They investigated light alkane dehydrogenation over single-Al sites in form of Z^−^[HGaX]^+^ (X = H, CH_3_, OH, Cl) and double-Al site in form of Z^2-^[GaH]^2+^over Ga-modified ZSM-5 and the correlation between the distance between framework Al and the activation energy on “Carbenium activation” pathway, and found that a more stable Ga site and a relatively smaller Al-Al distance, would result in a larger C-H activation energy barrier and a smaller energy barrier for H_2_ desorption and site regeneration. They concluded that the energy barrier can be controlled with the framework Al-Al distance [119].

**Figure 23:**
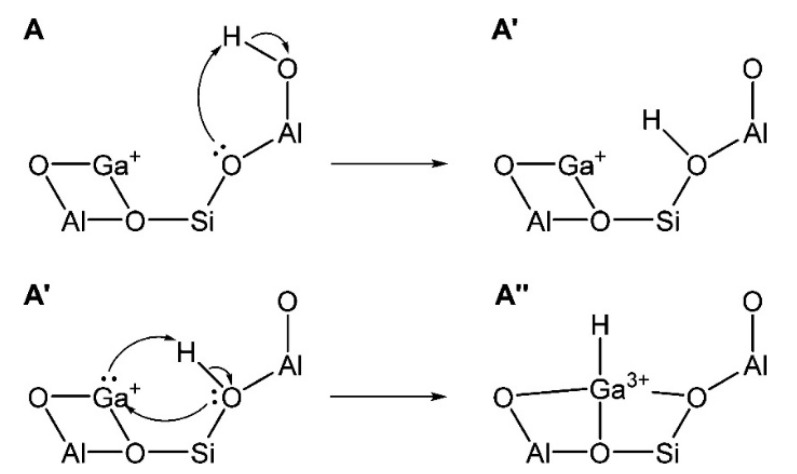
Two steps involved in the protonation of the Ga^+^ by the BAS to form the active [GaH]^2+^. Reprinted with permission from ref [73]. Copyright 2018 American Chemical Society.

**Figure 24:**
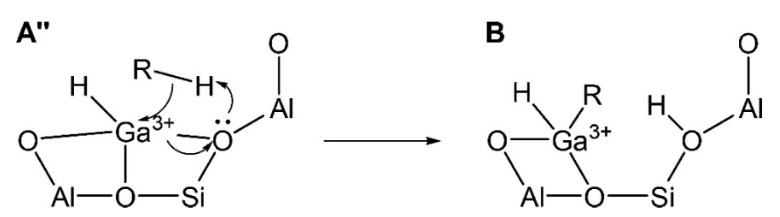
Alkane addition to the LBAS. Reprinted with permission from ref [73]. Copyright 2018 American Chemical Society. (Moritz W. Schreiber, 2018 [73]).

**Figure 25:**
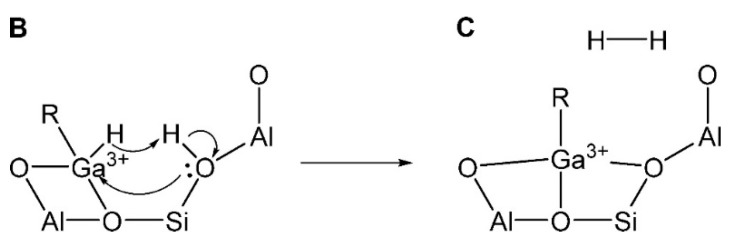
H_2_ elimination from the LBAS. Reprinted with permission from ref [73]. Copyright 2018 American Chemical Society.

The synergy between Ga species and adjacent BAS is apparent in view of the reported experimental performance [130]. The observed distribution of reaction products is also dependent on the ratio of Ga species to BAS, and imply that the BAS in ZSM-5 may also play a role in activation and oligomerization of formed olefins or act in concert with the Ga-species forming a bi-functional active site [89,99,100]. Iglesia et al. suggested that alkane activation took place at acidic sites and Ga species are the reaction sites for H_2_ recombination and desorption [134]. Conte and coworkers proposed that Ga species may coordinate with olefins, interact with adjacent acid sites and may promote methanol to aromatics process [135]. Experiments have shown that when all the BAS in Ga-modified ZSM-5 is neutralized with pyridine, the catalyst activity is greatly reduced or even completely deactivated. This indicates that the conversion process of light hydrocarbons requires the synergetic interaction between BAS and LAS, including the Ga species [135]. Gao identified and quantified the coordination sites of Ga species and synergy between LAS and BAS by ^1^H-^71^Ga double resonance solid-state NMR, and proposed the synergy of Ga species and BAS site in the dehydrogenation and aromatization of alkanes [123]. Pidko et al. showed that Ga species functions as LAS in ethane activation and promotes the cleavage of C-H bond. They also showed that ethane dehydrogenation was significantly promoted when BAS and Ga sites were very close [113]. Schreiber et al. proposed according to reaction kinetics that, the alkane dehydrogenation rate is the highest when Ga/Al = 1:2 and Ga/BAS = 1:1. The subsequent DFT calculations compared the apparent activation energy of the catalyst with only LAS or BAS, and both LAS and BAS. According to the calculated reaction barriers, the participation of both, BAS and LAS may lead to propane C-H bond activation with lower activation energy barrier (Figure 23, Figure 24 and Figure 25) [73].

**Figure 26:**
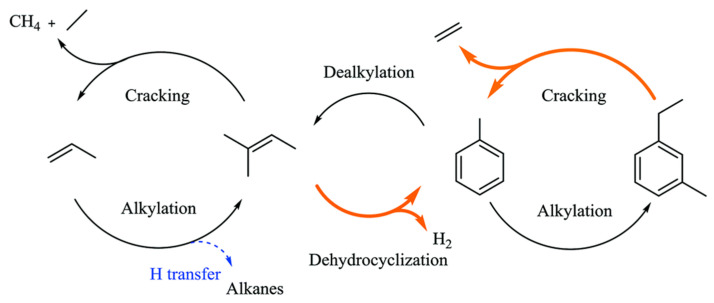
Proposed Ga role for ethanol conversion over Ga-ZSM-5: orange lines for promoted reactions and blue dashed line for suppressed reaction. Reprinted with permission from ref [49]. Copyright 2017 Royal Society of Chemistry.

He et al. showed that propane dehydrogenation and aromatization can only take place with LAS-BAS bi-functional Ga species, aromatics are only generated at the bi-functional sites [74,103,136]. Li et al. proposed that Ga species promotes dehydrogenation and prohibits hydrogen transfer for formation of light alkanes, and thus, benefits the formation of aromatics. The “promoted” and “suppressed” pathways are shown in Figure 26 [49].

People also investigated the synergy effect of adjacent BAS to the catalytic performance of the LAS-BAS pair, by combining the experimental and theoretical efforts. Phadke et al. investigated the free energy landscape for the dehydrogenation pathway over Ga^+^-H^+^ cation pairs. They showed that Gibbs free energy barrier of the rate-determining step of alkyl activation over [GaH]^2+^ is ~20 kcal/mol lower than concerted elimination of C_3_H_6_ and H_2_ from [C_3_H_7_-GaH]^+^-H^+^ cation pairs, while the formation of [GaH_2_]^+^-H^+^ cation pairs from [GaH]^2+^ cations is thermodynamically and kinetically feasible. In this sense, the dehydrogenation of propane over [GaH_2_]^+^-H^+^ cation pairs would be much less favorable than those involving [GaH]^2+^ cations. [GaH_2_]^+^-H^+^ cation pairs can activate C_3_H_8_ to produce [C_3_H_7_-GaH]^+^-H^+^ cation pairs and the barrier for formation of [GaH]^2+^ cations from [C_3_H_7_-GaH]^+^-H^+^ cation pairs is much lower than that to regenerate [GaH_2_]^+^-H^+^ cation pairs. Therefore, they proposed that [GaH]^2+^ cations are the primary active sites responsible for dehydrogenation [52,83].

**Figure 27:**
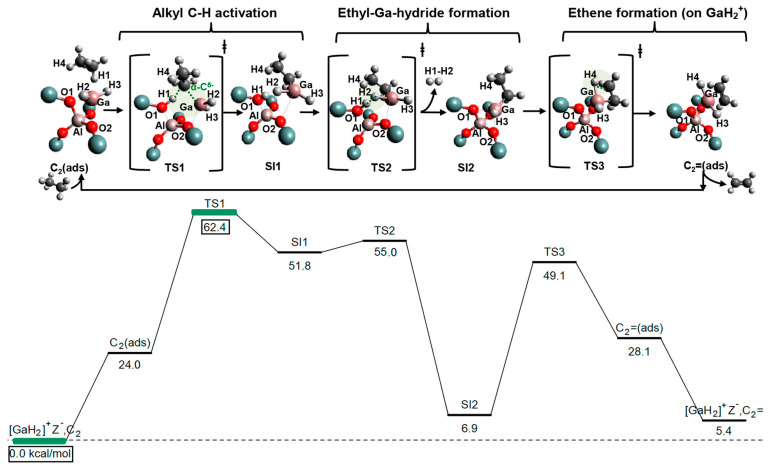
Ethane dehydrogenation via a stepwise alkyl mechanism on [GaH_2_]^+^ and the corresponding free energy surface (kcal/mol) reported at 823 K and 1 atm. Reprinted with permission from ref [94]. Copyright 2018 American Chemical Society.

**Figure 28:**
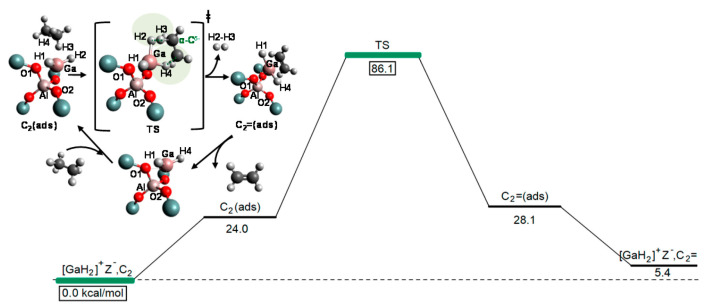
Ethane dehydrogenation via the concerted mechanism on [GaH_2_]^+^ and the corresponding free energy surface (kcal/mol) reported at 823 K and 1 atm. Reprinted with permission from ref [94]. Copyright 2018 American Chemical Society.

**Figure 29:**
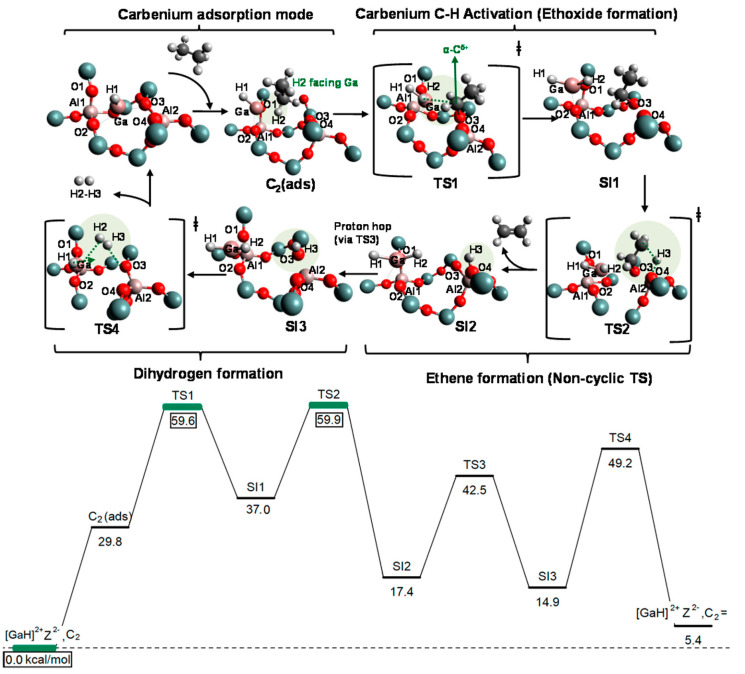
Ethane dehydrogenation via the carbenium mechanism (noncyclic route) on [GaH]^2+^ (configuration B), and the corresponding free energy surface (kcal/mol), reported at 823 K and 1 atm. Reprinted with permission from ref [94]. Copyright 2018 American Chemical Society.

Mansoor et al. compared the mechanisms for light alkanes dehydrogenation over Ga species, namely Ga^+^, [GaH]^2+^ and [GaH_2_]^+^, in ZSM-5 (Figure 27, Figure 28 and Figure 29). They proposed that, for ethane dehydrogenation, the stepwise alkyl activation is favored over [GaH_2_]^+^ with a calculated apparent free energy barrier (ΔG_app_^‡^) of 62.4 kcal/mol and is 23.7 kcal/mol lower than that for the alkyl activation through concerted mechanism. The calculated ΔG_app_^‡^ for ethane dehydrogenation over [GaH]^2+^ through carbenium and alkyl activation are 60.9 and 69.3 kcal/mol, respectively, suggesting the preference for carbenium mechanism. The small difference in ΔG_app_^‡^ also suggest that both [GaH_2_]^+^ and [GaH]^2+^ are relevant to the reaction kinetics. As the ΔG_app_^‡^ suggests that the formation of [GaH_2_]^+^ and [GaH]^2+^ are kinetically favored from alkyl Ga hydride, Ga^+^ may be not effective for C-H activation and would evolve into [GaH_2_]^+^ in reaction conditions. They also investigated the dehydrogenation of propane and attributed the decrease of activation enthalpy to the electron-donating group attached to β-C [83,94].

Krishnamurthy et al. studied the reaction kinetics of propane dehydroaromatization, considering two gallium species, [GaH]^2+^ and [GaH_2_]^+^. The results show that active site for propane dehydroaromatization on Ga modified HZSM-5 varies with Ga loading and Si/Al ratio. [GaH]^2+^ is formed as dominant reaction site. When Ga/Al is high, [GaH]^2+^ is transformed to [GaH_2_]^+^ that is highly active [42]. Thivasasith et al. investigated *n*-hexane dehydroaromatization to benzene over Ga^+^ and [GaH_2_]^+^. They showed that the reaction proceeds with dehydrogenation of *n*-hexane to hexa-1,3,5-triene and dehydroaromatization of hexa-1,3,5-triene to benzene. Hexane dehydrogenation is the rate-determining step over Ga^+^ with an activation barrier of 76.6 kcal mol^−1^, while the hydrogen abstraction from n-hexane is the rate-limiting step with a barrier of 11.1 kcal mol^−1^ over [GaH_2_]^+^. The findings suggest the existence of [GaH_2_]^+^ as one of the most active species for the dehydroaromatization of alkanes [92].

These works highlight both the intrinsic activity of Ga-based LAS acting in synergy with BAS to the dehydrogenation of light alkanes, based on the evolution of Ga species with the BAS in reaction conditions. However, the formation of highly reactive Ga species, such as [GaH]^2+^ and [GaH_2_]^+^-H^+^ cation pair, depend strongly on BAS in reasonable proximity from the Ga-based LAS, and may require well-controlled synthetic routes.

## 5. Conclusions

The recent experimental and theoretical efforts demonstrated the high efficiency and stability of Ga-modified ZSM-5 in the dehydrogenation of light hydrocarbons to produce olefins and aromatics. Ga-modified ZSM-5 can be prepared by ion exchange, incipient wetness impregnation, chemical vapor deposition, etc. Compared with pure MFI-type zeolite, Ga-modified ZSM-5 exhibits superior catalytic performance in conversion of light alkanes. The observed performance of Ga-modified ZSM-5 can be attributed to formation of various Ga-species that efficiently catalyze the dehydrogenation and dehydroaromatization of light alkanes. Previous experimental findings suggest the catalytic performance of Ga-modified ZSM-5 depend strongly on the detailed preparation methods, the pre-treatment and reaction conditions. Recent investigations with in-situ and off-situ techniques, as well as theoretical investigations, also suggest the potential evolution of Ga species in reaction conditions. However, the formation and evolution of Ga species in reaction conditions, and their contribution to the conversion of light alkanes, as well as coke deposition, are not well-rationalized, and more insights from newly developed in-situ and operando characterization techniques and theoretical approaches would be highly desired. In addition to these, controlled synthesis of zeolites with desired Al distribution and well-defined deposition of Ga species would be necessary for fabrication of the proposed highly-active Ga-modified ZSM-5 for catalytic applications. Further investigations on these aspects would guide the controlled fabrication of Ga-modified ZSM-5 with expected catalytic performance and benefit the development of novel catalysts for efficient conversion of light alkanes to value-added chemicals.

## Data Availability

Not applicable.

## References

[1] Breck D.W. (1964). Crystalline molecular sieves. J. Chem. Educ..

[2] Meier W.M., Olson D.H., Baerlocher C. (1996). Atlas of zeolite structure types. Zeolites.

[3] Gayubo A.G., Aguayo A.T., Atutxa A., Prieto R., Bilbao J. (2004). Deactivation of a HZSM-5 zeolite catalyst in the transformation of the aqueous fraction of biomass pyrolysis oil into hydrocarbons. Energy Fuels.

[4] Taifan W., Baltrusaitis J. (2016). CH_4_ conversion to value added products: Potential, limitations and extensions of a single step heterogeneous catalysis. Appl Catal. B Environ..

[5] Lunsford J.H. (2000). Catalytic conversion of methane to more useful chemicals and fuels: A challenge for the 21st century. Catal. Today.

[6] Park J.H., Lee D.W., Im S.W., Lee Y.H., Suh D.J., Jun K.W., Lee K.Y. (2012). Oxidative coupling of methane using non-stoichiometric lead hydroxyapatite catalyst mixtures. Fuel.

[7] Guisnet M., Gnep N.S., Alario F. (1992). Aromatization of short chain alkanes on zeolite catalysts. Appl. Catal. A Gen..

[8] Jin M.H., Lee C.B., Lee D.W., Lee S.W., Park J.W., Oh D., Hwang K.R., Lee K.Y., Park J.S. (2016). Microchannel methane steam reformers with improved heat transfer efficiency and their long-term stability. Fuel.

[9] Gim M.Y., Song C., Kim T.H., Song J.H., Kim D.H., Lee K.Y., Song I.K. (2017). BTX production by coaromatization of methane and propane over gallium oxide supported on mesoporous HZSM-5. Mol. Catal..

[10] Qiu B., Jiang F., Lu W.D., Yan B., Li W.C., Zhao Z.C., Lu A.H. (2020). Oxidative dehydrogenation of propane using layered borosilicate zeolite as the active and selective catalyst. J. Catal..

[11] Michorczyk P., Zenczak-Tomera K., Michorczyk B., Wegrzyniak A., Basta M., Millot Y., Valentin L., Dzwigaj S. (2020). Effect of dealumination on the catalytic performance of Cr-containing Beta zeolite in carbon dioxide assisted propane dehydrogenation. J. Co_2_ Util..

[12] Liang B., Zhang X., Xie Y., Lin R.B., Krishna R., Cui H., Li Z., Shi Y., Wu H., Zhou W. (2020). An Ultramicroporous Metal-Organic Framework for High Sieving Separation of Propylene from Propane. J. Am. Chem. Soc..

[13] Amghizar I., Vandewalle L.A., Van Geem K.M., Marin G.B. (2017). New Trends in Olefin Production. Engineering.

[14] Kosinov N., Hensen E.J.M. (2020). Reactivity, Selectivity, and Stability of Zeolite-Based Catalysts for Methane Dehydroaromatization. Adv. Mater..

[15] Bjørgen M., Joensen F., Spangsberg Holm M., Olsbye U., Lillerud K.-P., Svelle S. (2008). Methanol to gasoline over zeolite H-ZSM-5: Improved catalyst performance by treatment with NaOH. Appl. Catal. A Gen..

[16] Bhasin M.M., McCain J.H., Vora B.V., Imai T., Pujado P.R. (2001). Dehydrogenation and oxydehydrogenation of paraffins to olefins. Appl. Catal. A Gen..

[17] Song H., Rioux R.M., Hoefelmeyer J.D., Komor R., Niesz K., Grass M., Yang P., Somorjai G.A. (2006). Hydrothermal growth of mesoporous SBA-15 silica in the presence of PVP-stabilized Pt nanoparticles: Synthesis, characterization, and catalytic properties. J. Am. Chem. Soc..

[18] Hu Z.P., Wang Z., Yuan Z.Y. (2020). Cr/Al_2_O_3_ catalysts with strong metal-support interactions for stable catalytic dehydrogenation of propane to propylene. Mol. Catal..

[19] Li G.N., Vollmer I., Liu C., Gascon J., Pidko E.A. (2019). Structure and Reactivity of the Mo/ZSM-5 Dehydroaromatization Catalyst: An Operando Computational Study. ACS Catal..

[20] Uslamin E.A., Saito H., Sekine Y., Hensen E.J.M., Kosinov N. (2020). Different mechanisms of ethane aromatization over Mo/ZSM-5 and Ga/ZSM-5 catalysts. Catal. Today.

[21] Ji Z.H., Lv H.F., Pan X.L., Bao X.H. (2018). Enhanced ethylene selectivity and stability of Mo/ZSM5 upon modification with phosphorus in ethane dehydrogenation. J. Catal..

[22] Hu Z.P., Chen C., Ren J.T., Yuan Z.Y. (2018). Direct dehydrogenation of propane to propylene on surface-oxidized multiwall carbon nanotubes. Appl. Catal. A Gen..

[23] Hu Z.P., Zhao H., Chen C., Yuan Z.Y. (2018). Castanea mollissima shell-derived porous carbons as metal-free catalysts for highly efficient dehydrogenation of propane to propylene. Catal. Today.

[24] Shimada H., Akazawa T., Ikenaga N., Suzuki T. (1998). Dehydrogenation of isobutane to isobutene with iron-loaded activated carbon catalyst. Appl. Catal. A Gen..

[25] Cheng Y.H., Lei T.Q., Miao C.X., Hua W.M., Yue Y.H., Gao Z. (2018). Ga_2_O_3_/NaZSM-5 for C2H6 dehydrogenation in the presence of CO_2_: Conjugated effect of silanol. Microporous Mesoporous Mater..

[26] Shen Z.H., Liu J., Xu H.L., Yue Y.H., Hua W.M., Shen W. (2009). Dehydrogenation of ethane to ethylene over a highly efficient Ga_2_O_3_/HZSM-5 catalyst in the presence of CO_2_. Appl. Catal. A Gen..

[27] Rioux R.M., Song H., Hoefelmeyer J.D., Yang P., Somorjai G.A. (2005). High-surface-area catalyst design: Synthesis, characterization, and reaction studies of platinum nanoparticles in mesoporous SBA-15 silica. J. Phys. Chem. B.

[28] Moulijn J.A., van Diepen A.E., Kapteijn F. (2001). Catalyst deactivation: Is it predictable?. Appl. Catal. A Gen..

[29] Weckhuysen B.M., Schoonheydt R.A. (1999). Alkane dehydrogenation over supported chromium oxide catalysts. Catal. Today.

[30] Wang N., Dong X., Liu L., Cai D., Cheng Q., Wang J., Hou Y., Emwas A.-H., Gascon J., Han Y. (2021). Probing the Catalytic Active Sites of Mo/HZSM-5 and Their Deactivation during Methane Dehydroaromatization. Cell Rep. Phys. Sci..

[31] Mokrani T., Scurrell M. (2009). Gas Conversion to Liquid Fuels and Chemicals: The Methanol Route-Catalysis and Processes Development. Catal. Rev..

[32] Xie S.B., Chen K.D., Bell A.T., Iglesia E. (2000). Structural characterization of molybdenum oxide supported on zirconia. J. Phys. Chem. B.

[33] Gnep N.S., Doyemet J.Y., Guisnet M. (1988). Role Of Gallium Species on the Dehydrocyclodimerization Of Propane on Zsm5 Catalysts. J. Mol. Catal..

[34] Hagen A., Roessner F. (2000). Ethane to aromatic hydrocarbons: Past, present, future. Catal. Rev..

[35] Biscardi J.A., Iglesia E. (1996). Structure and function of metal cations in light alkane reactions catalyzed by modified H-ZSM5. Catal. Today.

[36] Iglesia E., Baumgartner J.E. (1993). Hydrogen-Transfer And Activation of Propane And Methane on Zsm5-Based Catalysts. Catal. Lett..

[37] Dooley K.M., Guidry T.F., Price G.L. (1995). Control of intrazeolitic gallium cation content and its effects on C2 dehydrogenation in Ga-MFI catalysts. J. Catal..

[38] Price G.L., Kanazirev V., Dooley K.M., Hart V.I. (1998). On the mechanism of propane dehydrocyclization over cation-containing, proton-poor MFI zeolite. J. Catal..

[39] Kazansky V.B., Subbotina I.R., Rane N., van Santen R.A., Hensen E.J. (2005). On two alternative mechanisms of ethane activation over ZSM-5 zeolite modified by Zn^2+^ and Ga^1+^ cations. Phys. Chem. Chem. Phys. PCCP.

[40] Fanchiang W.L., Lin Y.C. (2012). Catalytic fast pyrolysis of furfural over H-ZSM-5 and Zn/H-ZSM-5 catalysts. Appl. Catal. A Gen..

[41] Bhan A., Delgass W.N. (2008). Propane aromatization over HZSM-5 and Ga/HZSM-5 catalysts. Catal. Rev..

[42] Krishnamurthy G., Bhan A., Delgass W.N. (2010). Identity and chemical function of gallium species inferred from microkinetic modeling studies of propane aromatization over Ga/HZSM-5 catalysts. J. Catal..

[43] Ono Y., Kitagawa H., Sendoda Y. (1987). Transformation Of but-1-Ene into Aromatic-Hydrocarbons over ZSM-5 Zeolites. J. Chem. Soc. Faraday T 1.

[44] Davies E.E., Kolombos A.J. (1979). Process for Converting C3-C12 Hydrocarbons to Aromatics over Gallia-Activated Zeolite. Google Patents.

[45] Song C., Gim M.Y., Lim Y.H., Kim D.H. (2019). Enhanced yield of benzene, toulene, and xylene from the co-aromatization of methane and propane over gallium supported on mesoporous ZSM-5 and ZSM-11. Fuel.

[46] Xin M.D., Xing E.H., Gao X.Z., Wang Y.R., Ouyang Y., Xu G.T., Luo Y.B., Shu X.T. (2019). Ga Substitution during Modification of ZSM-5 and Its Influences on Catalytic Aromatization Performance. Ind. Eng. Chem. Res..

[47] Hsieh C.Y., Chen Y.Y., Lin Y.C. (2018). Ga-Substituted Nanoscale HZSM-5 in Methanol Aromatization: The Cooperative Action of the Bronsted Acid and the Extra-Framework Ga Species. Ind. Eng. Chem. Res..

[48] Cybulskis V.J., Pradhan S.U., Lovon-Quintana J.J., Hock A.S., Hu B., Zhang G.H., Delgass W.N., Ribeiro F.H., Miller J.T. (2017). The Nature of the Isolated Gallium Active Center for Propane Dehydrogenation on Ga/SiO_2_. Catal. Lett..

[49] Li Z.L., Lepore A.W., Salazar M.F., Foo G.S., Davison B.H., Wu Z.L., Narula C.K. (2017). Selective conversion of bio-derived ethanol to renewable BTX over Ga-ZSM-5. Green Chem..

[50] Lai P.C., Chen C.H., Hsu H.Y., Lee C.H., Lin Y.C. (2016). Methanol aromatization over Ga-doped desilicated HZSM-5. RSC Adv..

[51] Rane N., Kersbulck M., van Santen R.A., Hensen E.J.M. (2008). Cracking of n-heptane over Bronsted acid sites and Lewis acid Ga sites in ZSM-5 zeolite. Microporous Mesoporous Mater..

[52] Phadke N.M., Van der Mynsbrugge J., Mansoor E., Getsoian A.B., Head-Gordon M., Bell A.T. (2018). Characterization of Isolated Ga^3+^ Cations in Ga/H-MFI Prepared by Vapor-Phase Exchange of H-MFI Zeolite with GaCl_3_. ACS Catal..

[53] Shao C.T., Lang W.Z., Yan X., Guo Y.J. (2017). Catalytic performance of gallium oxide based-catalysts for the propane dehydrogenation reaction: Effects of support and loading amount. RSC Adv..

[54] Han Z., Zhou F., Liu Y., Qiao K., Wu G. (2019). Synthesis of gallium-containing ZSM-5 zeolites by the seed-induced method and catalytic performance of GaZSM-5 and AlZSM-5 during the conversion of methanol to olefins. J. Taiwan Inst. Chem. Eng..

[55] Su X.F., Wang G.L., Bai X.F., Wu W., Xiao L.F., Fang Y.J., Zhang J.W. (2016). Synthesis of nanosized HZSM-5 zeolites isomorphously substituted by gallium and their catalytic performance in the aromatization. Chem. Eng. J..

[56] Li J., Yu Y.Q., Li X.Y., Wang W., Yu G., Deng S.B., Huang J., Wang B., Wang Y.J. (2015). Maximizing carbon efficiency of petrochemical production from catalytic co-pyrolysis of biomass and plastics using gallium-containing MFI zeolites. Appl. Catal. B Environ..

[57] Raad M., Hamieh S., Toufaily J., Hamieh T., Pinard L. (2018). Propane aromatization on hierarchical Ga/HZSM-5 catalysts. J. Catal..

[58] Wannapakdee W., Suttipat D., Dugkhuntod P., Yutthalekha T., Thivasasith A., Kidkhunthod P., Nokbin S., Pengpanich S., Limtrakul J., Wattanakit C. (2019). Aromatization of C5 hydrocarbons over Ga-modified hierarchical HZSM-5 nanosheets. Fuel.

[59] Kosslick H., Richter M., Tuan V.A., Parlitz B., Szulzewsky K., Fricke R. (1991). Genesis of gallosilicates with ZSM-5 structure. Insertion of Ga and zeolitic properties at various steps of crystallization. Studies in Surface Science and Catalysis.

[60] Nishi K., Komai S., Inagaki K., Satsuma A., Hattori T. (2002). Structure and catalytic properties of Ga-MFI in propane aromatization. Appl. Catal. A Gen..

[61] Choudhary V.R., Banerjee S., Panjala D. (2002). Product distribution in the aromatization of dilute ethene over H-GaAlMFI zeolite: Effect of space velocity. Microporous Mesoporous Mater..

[62] Al-Yassir N., Akhtar M.N., Al-Khattaf S. (2011). Physicochemical properties and catalytic performance of galloaluminosilicate in aromatization of lower alkanes: A comparative study with Ga/HZSM-5. J. Porous Mater..

[63] Wannapakdee W., Wattanakit C., Paluka V., Yutthalekha T., Limtrakul J. (2016). One-pot synthesis of novel hierarchical bifunctional Ga/HZSM-5 nanosheets for propane aromatization. RSC Adv..

[64] Kim W.G., So J., Choi S.W., Liu Y.J., Dixit R.S., Sievers C., Sholl D.S., Nair S., Jones C.W. (2017). Hierarchical Ga-MFI Catalysts for Propane Dehydrogenation. Chem. Mater..

[65] Awate S.V., Joshi P.N., Shiralkar V.P., Kotasthane A.N. (1992). Synthesis And Characterization Of Gallosilicate Pentasil (MFI) Framework Zeolites. J. Incl. Phenom. Mol..

[66] Montes A., Giannetto G. (2000). A new way to obtain acid or bifunctional catalysts V. Considerations on bifunctionality of the propane aromatization reaction over [Ga,Al]-ZSM-5 catalysts. Appl. Catal. A Gen..

[67] Choudhary V.R., Banerjee S., Panjala D. (2002). Influence of Temperature on the Product Selectivity and Distribution of Aromatics and C8 Aromatic Isomers in the Conversion of Dilute Ethene over H-Galloaluminosilicate (ZSM-5 type) Zeolite. J. Catal..

[68] Choudhary V.R., Panjala D., Banerjee S. (2002). Aromatization of propene and n-butene over H-galloaluminosilicate (ZSM-5 type) zeolite. Appl. Catal. A Gen..

[69] Choudhary T.V., Kinage A., Banerjee S., Choudhary V.R. (2006). Propane Conversion to Aromatics on Highly Active H-GaAlMFI:  Effect of Thermal Pretreatment. Energy Fuels.

[70] Raad M., Astafan A., Hamieh S., Toufaily J., Hamieh T., Comparot J.D., Canaff C., Daou T.J., Patarin J., Pinard L. (2018). Catalytic properties of Ga-containing MFI-type zeolite in cyclohexane dehydrogenation and propane aromatization. J. Catal..

[71] Kosslick H., Tuan V.A., Parlitz B., Fricke R., Peuker C., Storek W. (1993). Disruption Of the Mfi Framework by the Incorporation of Gallium. J. Chem. Soc. Faraday T.

[72] Kosslick H., Tuan V.A., Fricke R. (1991). Ga–ZSM–5–Conditions of Synthesis. Cryst. Res. Technol..

[73] Schreiber M.W., Plaisance C.P., Baumgartl M., Reuter K., Jentys A., Bermejo-Deval R., Lercher J.A. (2018). Lewis-Bronsted Acid Pairs in Ga/H-ZSM-5 To Catalyze Dehydrogenation of Light Alkanes. J. Am. Chem. Soc..

[74] Choudhary V.R., Mantri K., Sivadinarayana C. (2000). Influence of zeolite factors affecting zeolitic acidity on the propane aromatization activity and selectivity of Ga/H-ZSM-5. Microporous Mesoporous Mater..

[75] Kazansky V., Subbotina I., Vansanten R., Hensen E. (2004). DRIFTS study of the chemical state of modifying gallium ions in reduced Ga/ZSM-5 prepared by impregnationI. Observation of gallium hydrides and application of CO adsorption as a molecular probe for reduced gallium ions. J. Catal..

[76] Kwak B.S., Sachtler W.M.H. (1994). Effect Of Ga/Proton Balance In Ga/Hzsm-5 Catalysts on C-3 Conversion To Aromatics. J. Catal..

[77] Nowak I. (2003). Effect of H_2_–O_2_ pre-treatments on the state of gallium in Ga/H-ZSM-5 propane aromatisation catalysts. Appl. Catal. A Gen..

[78] Kanazirev V., Price G.L., Dooley K.M. (1990). Enhancement in Propane Aromatization with Ga_2_O_3_/HZSM-5 Catalysts. J. Chem. Soc. Chem. Comm..

[79] Freeman D., Wells R.P., Hutchings G.J. (2001). Methanol to hydrocarbons: Enhanced aromatic formation using a composite Ga_2_O_3_-H-ZSM-5 catalyst. Chem. Commun..

[80] Freeman D., Wells R.P.K., Hutchings G.J. (2002). Conversion of methanol to hydrocarbons over Ga_2_O_3_/H-ZSM-5 and Ga_2_O_3_/WO_3_ catalysts. J. Catal..

[81] Garciasanchez M. (2003). Characterization of Ga/HZSM-5 and Ga/HMOR synthesized by chemical vapor deposition of trimethylgallium. J. Catal..

[82] Hensen E.J.M., García-Sánchez M., Rane N., Magusin P.C.M.M., Liu P.-H., Chao K.-J., van Santen R.A. (2005). In situ Ga K edge XANES study of the activation of Ga/ZSM-5 prepared by chemical vapor deposition of trimethylgallium. Catal. Lett..

[83] Phadke N.M., Mansoor E., Bondil M., Head-Gordon M., Bell A.T. (2019). Mechanism and Kinetics of Propane Dehydrogenation and Cracking over Ga/H-MFI Prepared via Vapor-Phase Exchange of H-MFI with GaCl_3_. J. Am. Chem. Soc..

[84] El-Malki E.M., van Santen R.A., Sachtler W.M.H. (1999). Introduction of Zn, Ga, and Fe into HZSM-5 cavities by sublimation: Identification of acid sites. J. Phys. Chem. B.

[85] Liu R.-l., Zhu H.-q., Wu Z.-w., Qin Z.-f., Fan W.-b., Wang J.-g. (2015). Aromatization of propane over Ga-modified ZSM-5 catalysts. J. Fuel Chem. Technol..

[86] Hamid S.B.A., Derouane E.G., Meriaudeau P., Naccache C. (1996). Effect of reductive and oxidative atmospheres on the propane aromatisation activity and selectivity of Ga/H-ZSM-5 catalysts. Catal. Today.

[87] Rodrigues V.D., Faro A.C. (2012). On catalyst activation and reaction mechanisms in propane aromatization on Ga/HZSM5 catalysts. Appl. Catal. A Gen..

[88] Arnaldo da Costa Faro Júniora A., de Oliveira Rodriguesa V. (2008). Pulse reaction studies of gallium modified H-ZSM5 catalysts with propane. Adv. Pharmacol..

[89] Ausavasukhi A., Sooknoi T. (2014). Tunable activity of [Ga]HZSM-5 with H_2_ treatment: Ethane dehydrogenation. Catal. Commun..

[90] Fricke R., Kosslick H., Lischke G., Richter M. (2000). Incorporation of gallium into zeolites: Syntheses, properties and catalytic application. Chem. Rev..

[91] Su X., Fang Y., Gao P., Liu Y., Hou G., Bai X., Wu W. (2020). In-situ microwave synthesis of nano-GaZSM-5 bifunctional catalysts with controllable location of active GaO+ species for olefins aromatization. Microporous Mesoporous Mater..

[92] Thivasasith A., Maihom T., Pengpanich S., Limtrakul J., Wattanakit C. (2019). Insights into the reaction mechanism of n-hexane dehydroaromatization to benzene over gallium embedded HZSM-5: Effect of H_2_ incorporated on active sites. Phys. Chem. Chem. Phys. PCCP.

[93] Pidko E.A., Hensen E.J.M., van Santen R.A. (2012). Self-organization of extraframework cations in zeolites. Proc. R. Soc. A Math. Phys. Eng. Sci..

[94] Mansoor E., Head-Gordon M., Bell A.T. (2018). Computational Modeling of the Nature and Role of Ga Species for Light Alkane Dehydrogenation Catalyzed by Ga/H-MFI. ACS Catal..

[95] Kuo Y.-T., Almansa G.A., Vreugdenhil B.J. (2018). Catalytic aromatization of ethylene in syngas from biomass to enhance economic sustainability of gas production. Appl. Energy.

[96] Meitzner G.D., Iglesia E., Baumgartner J.E., Huang E.S. (1993). The Chemical State of Gallium in Working Alkane Dehydrocyclodimerization Catalysts. In situ Gallium K-Edge X-Ray Absorption Spectroscopy. J. Catal..

[97] Lopez-Sanchez J.A., Conte M., Landon P., Zhou W., Bartley J.K., Taylor S.H., Carley A.F., Kiely C.J., Khalid K., Hutchings G.J. (2012). Reactivity of Ga_2_O_3_ Clusters on Zeolite ZSM-5 for the Conversion of Methanol to Aromatics. Catal. Lett..

[98] Sirotin S.V., Moskovskaya I.F., Romanovsky B.V. (2011). Synthetic strategy for Fe-MCM-41 catalyst: A key factor for homogeneous or heterogeneous phenol oxidation. Catal. Sci. Technol..

[99] Michorczyk P. (2003). Dehydrogenation of propane to propene over gallium oxide in the presence of CO_2_. Appl. Catal. A Gen..

[100] Zheng B., Hua W., Yue Y., Gao Z. (2005). Dehydrogenation of propane to propene over different polymorphs of gallium oxide. J. Catal..

[101] Michorczyk P., Kuśtrowski P., Kolak A., Zimowska M. (2013). Ordered mesoporous Ga_2_O_3_ and Ga_2_O_3_–Al2O3 prepared by nanocasting as effective catalysts for propane dehydrogenation in the presence of CO_2_. Catal. Commun..

[102] Xu B., Li T., Zheng B., Hua W., Yue Y., Gao Z. (2007). Enhanced Stability of HZSM-5 Supported Ga_2_O_3_ Catalyst in Propane Dehydrogenation by Dealumination. Catal. Lett..

[103] Xiao H., Zhang J.F., Wang P., Zhang Z.Z., Zhang Q.D., Xie H.J., Yang G.H., Han Y.Z., Tan Y.S. (2015). Mechanistic insight to acidity effects of Ga/HZSM-5 on its activity for propane aromatization. RSC Adv..

[104] Al-Yassir N., Akhtar M.N., Ogunronbi K., Al-Khattaf S. (2012). Synthesis of stable H-galloaluminosilicate MFI with hierarchical pore architecture by surfactant-mediated base hydrolysis, and their application in propane aromatization. J. Mol. Catal. A Chem..

[105] Rodrigues V.d.O., Vasconcellos F.J., Faro Júnior A.d.C. (2016). Mechanistic studies through H–D exchange reactions: Propane aromatization in HZSM5 and Ga/HZSM5 catalysts. J. Catal..

[106] Xiao H., Zhang J., Wang X., Zhang Q., Xie H., Han Y., Tan Y. (2015). A highly efficient Ga/ZSM-5 catalyst prepared by formic acid impregnation and in situ treatment for propane aromatization. Catal. Sci. Technol..

[107] Lee B.J., Hur Y.G., Kim D.H., Lee S.H., Lee K.-Y. (2019). Non-oxidative aromatization and ethylene formation over Ga/HZSM-5 catalysts using a mixed feed of methane and ethane. Fuel.

[108] Ogunronbi K.E., Al-Yassir N., Al-Khattaf S. (2015). New insights into hierarchical metal-containing zeolites; synthesis and kinetic modelling of mesoporous gallium-containing ZSM-5 for propane aromatization. J. Mol. Catal. A Chem..

[109] Kazansky V., Subbotina I., Vansanten R., Hensen E. (2005). DRIFTS study of the nature and chemical reactivity of gallium ions in Ga/ZSM-5II. Oxidation of reduced Ga species in ZSM-5 by nitrous oxide or water. J. Catal..

[110] Faro Júnior A.d.C., Oliveira Rodrigues V.d., Gédéon A., Massiani P., Babonneau F. (2008). Pulse reaction studies of gallium modified H-ZSM5 catalysts with propane. Studies in Surface Science and Catalysis.

[111] Serykh A.I., Kolesnikov S.P. (2011). On the nature of gallium species in gallium-modified mordenite and MFI zeolites. A comparative DRIFT study of carbon monoxide adsorption and hydrogen dissociation. Phys. Chem. Chem. Phys..

[112] Rane N., Overweg A.R., Kazansky V.B., Santen R.A.V., Hensen E.J.M. (2006). Characterization and reactivity of Ga^+^ and GaO^+^ cations in zeolite ZSM-5. J. Catal..

[113] Pidko E.A., Kazansky V.B., Hensen E.J.M., van Santen R.A. (2006). A comprehensive density functional theory study of ethane dehydrogenation over reduced extra-framework gallium species in ZSM-5 zeolite. J. Catal..

[114] Pidko E.A., Hensen E.J.M., van Santen R.A. (2008). Anionic Oligomerization of Ethylene over Ga/ZSM-5 Zeolite: A Theoretical Study. J. Phys. Chem. C.

[115] Rodrigues V.D., Eon J.G., Faro A.C. (2010). Correlations between Dispersion, Acidity, Reducibility, and Propane Aromatization Activity of Gallium Species Supported on HZSM5 Zeolites. J. Phys. Chem. C.

[116] Pidko E.A., Hensen E.J.M., van Santen R.A. (2007). Dehydrogenation of light alkanes over isolated gallyl ions in Ga/ZSM-5 zeolites. J. Phys. Chem. C.

[117] Frash M.V., Van Santen R.A. (2000). Activation of small alkanes on Ga-exchanged zeolites: A quantum-chemical study of ethane dehydrogenation. J. Phys. Chem. A.

[118] Wongnongwa Y., Kidkhunthod P., Sukkha U., Pengpanich S., Thavornprasert K.-a., Phupanit J., Kungwan N., Feng G., Keawin T., Jungsuttiwong S. (2020). Local structure elucidation and reaction mechanism of light naphtha aromatization over Ga embedded H-ZSM-5 zeolite: Combined DFT and experimental study. Microporous Mesoporous Mater..

[119] Joshi Y.V., Thomson K.T. (2005). The roles of gallium hydride and Bronsted acidity in light alkane dehydrogenation mechanisms using Ga-exchanged HZSM-5 catalysts: A DFT pathway analysis. Catal. Today.

[120] Pereira M.S., Chaer Nascimento M.A. (2006). Theoretical study on the dehydrogenation reaction of alkanes catalyzed by zeolites containing nonframework gallium species. J. Phys. Chem. B.

[121] Chao K.-J., Liu P.-H. (2005). Gallium-containing zeolites: Characterization of catalytic role of gallium species in converting light paraffins to aromatics. Catal. Surv. Asia.

[122] Gao P., Xu J., Qi G., Wang C., Wang Q., Zhao Y., Zhang Y., Feng N., Zhao X., Li J. (2018). A Mechanistic Study of Methanol-to-Aromatics Reaction over Ga-Modified ZSM-5 Zeolites: Understanding the Dehydrogenation Process. ACS Catal..

[123] Gao P., Wang Q., Xu J., Qi G., Wang C., Zhou X., Zhao X., Feng N., Liu X., Deng F. (2018). Brønsted/Lewis Acid Synergy in Methanol-to-Aromatics Conversion on Ga-Modified ZSM-5 Zeolites, As Studied by Solid-State NMR Spectroscopy. ACS Catal..

[124] Hensen E.J., Pidko E.A., Rane N., van Santen R.A. (2007). Water-promoted hydrocarbon activation catalyzed by binuclear gallium sites in ZSM-5 zeolite. Angew. Chem..

[125] Jr A.C.F., Rodrigues V.D.O., Eon J.G. (2011). In Situ X-ray Absorption Study of the Genesis and Nature of the Reduced Gallium Species in Ga/HZSM5 Catalysts. J. Phys. Chem. C.

[126] Fang Y.J., Su X.F., Bai X.F., Wu W., Wang G.L., Xiao L.F., Yu A.R. (2017). Aromatization over nanosized Ga-containing ZSM-5 zeolites prepared by different methods: Effect of acidity of active Ga species on the catalytic performance. J. Energy Chem..

[127] Uslamin E.A., Luna-Murillo B., Kosinov N., Bruijnincx P.C.A., Pidko E.A., Weckhuysen B.M., Hensen E.J.M. (2019). Gallium-promoted HZSM-5 zeolites as efficient catalysts for the aromatization of biomass-derived furans. Chem. Eng. Sci..

[128] Broclawik E., Himei H., Yamadaya M., Kubo M., Miyamoto A., Vetrivel R. (1995). Density-functional theory calculations of the reaction pathway for methane activation on a gallium site in metal exchanged ZSM-5. J. Chem. Phys..

[129] Gomez E., Yan B., Kattel S., Chen J.G. (2019). Carbon dioxide reduction in tandem with light-alkane dehydrogenation. Nat. Rev. Chem..

[130] Guisnet M., Gnep N.S. (1996). Aromatization of propane over GaHMFI catalysts. Reaction scheme, nature of the dehydrogenating species and mode of coke formation. Catal. Today.

[131] Bandiera J., Ben Taarit Y. (1997). Ethane conversion: Kinetic evidence for the competition of consecutive steps for the same active centre. Appl. Catal. A Gen..

[132] Pereira M.S., Nascimento M.A.C. (2005). Theoretical study of the dehydrogenation reaction of ethane catalyzed by zeolites containing non-framework gallium species: The 3-step mechanism×the 1-step concerted mechanism. Chem. Phys. Lett..

[133] Pereira M.S., da Silva A.M., Chaer Nascimento M.A. (2011). Effect of the Zeolite Cavity on the Mechanism of Dehydrogenation of Light Alkanes over Gallium-Containing Zeolites. J. Phys. Chem. C.

[134] Jones A.J., Iglesia E. (2015). The Strength of Brønsted Acid Sites in Microporous Aluminosilicates. ACS Catal..

[135] Conte M., Lopez-Sanchez J.A., He Q., Morgan D.J., Ryabenkova Y., Bartley J.K., Carley A.F., Taylor S.H., Kiely C.J., Khalid K. (2012). Modified zeolite ZSM-5 for the methanol to aromatics reaction. Catal. Sci. Technol..

[136] Choudhary V.R., Devadas P., Banerjee S., Kinage A.K. (2001). Aromatization of dilute ethylene over Ga-modified ZSM-5 type zeolite catalysts. Microporous Mesoporous Mater..

